# Organelle-Selective Autophagy in Cancer Immunotherapy: Mitophagy and Nucleophagy-Related Nuclear Quality Control as Regulators of Tumor–Immune Interactions

**DOI:** 10.3390/ijms27198775

**Published:** 2026-09-30

**Authors:** Ayhan Bilir, Berna Yıldırım, Mete Hakan Karalök

**Affiliations:** 1 Department of Histology and Embryology, Faculty of Medicine, Istanbul Atlas University, Istanbul 34403, Türkiye; 2 Department of Obstetrics and Gynecology, Faculty of Medicine, Istanbul Atlas University, Istanbul 34403, Türkiye; hakan.karalok@atlas.edu.tr

**Keywords:** organelle-selective autophagy, mitophagy, nucleophagy, cancer immunotherapy, tumor immune evasion, mitochondrial DNA, micronuclei, cGAS–STING, nuclear quality control, immune-signal editing

## Abstract

Organelle-selective autophagy is an important regulator of tumor adaptation, immune signaling, and therapeutic resistance. By directing specific intracellular cargos to lysosomal degradation, selective autophagy pathways determine whether organelle damage is eliminated or remains available to innate immune sensors. In cancer, this is particularly relevant because mitochondrial and nuclear damage can generate immunologically active signals that influence tumor–immune interactions. This review examines mitophagy and nucleophagy-related nuclear quality-control mechanisms as regulators of cancer immunity and immunotherapy response. Mitophagy can limit mitochondrial ROS and mtDNA release while supporting metabolic adaptation, whereas nuclear quality-control mechanisms, including lamin B1 degradation, micronuclear clearance, and cGAS-associated micronucleophagy, may regulate the persistence of nuclear-derived DNA signals. We propose the concept of “intracellular immune-signal editing” to describe how organelle-selective autophagy controls the persistence, accessibility, and duration of mitochondrial and nuclear danger signals. The immunological outcome is highly context- and compartment-dependent because autophagy may protect tumor cells while supporting mitochondrial fitness and effector function in immune cells. We also discuss therapeutic implications, biomarkers, and experimental strategies required to distinguish true organelle-selective autophagic flux from static marker changes.

## 1. Introduction: Organelle Quality Control as a New Layer of Cancer Immune Regulation

Cancer immunotherapy has transformed oncology by demonstrating that durable tumor control can be achieved through the restoration or amplification of antitumor immune responses. Immune checkpoint inhibitors targeting programmed cell death protein 1 (PD-1), programmed death-ligand 1 (PD-L1), and cytotoxic T-lymphocyte-associated protein 4 (CTLA-4) have produced clinically meaningful responses across multiple malignancies. Nevertheless, only a subset of patients achieves durable benefit, and primary and acquired resistance remain major therapeutic challenges [1,2]. Resistance to immune checkpoint blockade has traditionally been attributed to tumor-intrinsic and tumor-extrinsic mechanisms, including defective antigen presentation, impaired interferon signaling, insufficient neoantigenicity, oncogenic pathway activation, immunosuppressive cytokines and cell populations, metabolic competition, and exclusion of cytotoxic lymphocytes from the tumor microenvironment [1,2]. However, these mechanisms do not fully explain how malignant cells continuously adapt to immune and therapeutic pressure at the level of intracellular organelles.

An emerging perspective is that tumor immune evasion is also shaped by intracellular quality-control systems that determine how cancer cells sense, compartmentalize, process, or eliminate stress-associated danger signals [3,4,5]. Tumor cells are chronically exposed to metabolic stress, hypoxia, oxidative injury, oncogene activation, DNA damage, proteotoxic stress, and anticancer therapies. These insults are registered within specific organelles, and the fate of damaged organelles can determine whether cellular stress promotes death, adaptation, senescence, inflammatory signaling, or immune recognition [3,4,5,6]. Organelle quality control may therefore represent an additional regulatory layer within tumor–immune interactions.

Selective autophagy is particularly relevant to this concept. Unlike bulk macroautophagy, selective autophagy recognizes defined cargos, including damaged organelles, protein aggregates, and nuclear or mitochondrial material, and directs them toward lysosomal degradation through cargo receptors and ATG8-family proteins such as LC3 and GABARAP [3,4,5]. In cancer, the consequences of this process are highly context-dependent. Selective autophagy can suppress malignant transformation by limiting oxidative and genomic damage, yet established tumors may exploit the same machinery to survive hypoxia, nutrient deprivation, therapy-induced injury, and immune-mediated stress [3,6,7]. Thus, selective autophagy can influence not only whether a cancer cell survives stress, but also whether that stress remains immunologically visible.

Mitophagy, the selective removal of damaged mitochondria, is the best-characterized example of this principle. PINK1/Parkin-dependent and receptor-mediated pathways involving BNIP3, NIX/BNIP3L, and FUNDC1 regulate mitochondrial turnover, reactive oxygen species (ROS), metabolic adaptation, and the availability of mitochondrial damage-associated molecular patterns [8,9,10]. Of particular relevance to tumor immunity, mitochondrial DNA (mtDNA) released from damaged mitochondria can activate cyclic GMP–AMP synthase–stimulator of interferon genes (cGAS–STING) signaling and downstream inflammatory responses [11,12,13]. Mitophagy therefore occupies a paradoxical position: efficient mitochondrial clearance can prevent excessive oxidative and inflammatory injury, but it may also reduce the persistence of mitochondrial danger signals capable of enhancing tumor immunogenicity. Conversely, insufficient mitochondrial quality control may increase innate immune activation while also promoting chronic inflammatory stress and genomic instability [8,11,12,13].

Nuclear quality control represents a less established but potentially analogous regulatory system. Compared with mitophagy, nucleophagy and related nuclear autophagy pathways remain incompletely defined in mammalian cancer cells. Nevertheless, accumulating evidence indicates that selected nuclear components can be processed through autophagy-related mechanisms. LC3 directly interacts with lamin B1 and mediates the degradation of nuclear lamina material during oncogenic stress, linking autophagy to nuclear remodeling and senescence [14,15]. In parallel, micronuclei generated by chromosomal instability, mitotic errors, or DNA damage can expose genomic DNA to cGAS and activate STING-dependent innate immune signaling [16,17]. Importantly, cGAS has also been reported to function as a micronucleophagy receptor, promoting autophagic clearance of micronuclei and limiting micronucleus-driven cGAMP production after genotoxic stress [18].

These observations suggest that mitochondrial and nuclear material should not be viewed simply as passive by-products of cellular damage. Their persistence, accessibility, and degradation can be actively regulated by organelle-selective autophagy, thereby influencing the magnitude and duration of intracellular danger signaling. In this review, we propose ‘intracellular immune-signal editing’ as a conceptual framework describing how cargo-specific organelle clearance may alter the persistence, accessibility, and duration of intracellular danger signals. This framework integrates established mechanisms with emerging and hypothesis-generating relationships and should not be interpreted as a single experimentally defined signaling pathway.

Recent reviews have comprehensively addressed the broader relationship between autophagy and cancer immunotherapy, including canonical autophagy signaling, pharmacological modulation, immune-cell function, and combination treatment strategies [19]. Other recent reviews have focused on individual selective autophagy pathways, whereas mammalian nucleophagy has increasingly been discussed as a distinct nuclear quality-control process involving cargos such as lamins, histones, DNA–protein crosslinks, micronuclei, and chromatin fragments [20]. The present review addresses a complementary but more specific question: how does selective clearance of damaged mitochondrial and nuclear-derived cargo regulate the persistence and accessibility of intracellular danger signals that influence tumor immune recognition?

Mitophagy and nucleophagy-related nuclear quality-control mechanisms are mechanistically distinct and should not be considered equivalent selective autophagy pathways. Nevertheless, they can converge functionally at the level of intracellular danger-signal availability. Damaged mitochondria can release mtDNA and mitochondrial stress signals, whereas micronuclei, ruptured nuclear structures, and chromatin fragments can expose genomic DNA to cytosolic sensors. Selective removal of these cargos can therefore alter the magnitude and duration of innate immune signaling, including cGAS–STING activation. Rather than implying that selective autophagy is uniformly immunostimulatory or immunosuppressive, this framework emphasizes which organelle-derived signal is generated, whether it is retained or degraded, in which cellular compartment this occurs, and for how long it remains accessible to immune-sensing pathways.

This distinction defines the scope of the present review. Rather than focusing broadly on mitochondrial or nuclear stress, we examine the selective recognition, sequestration, degradation, and immune consequences of organelle-specific damage. We first outline the molecular principles of selective autophagy and then discuss mitophagy as a regulator of mitochondrial danger signaling and immunotherapy response. We subsequently examine nucleophagy-related processes, including nuclear lamina degradation, micronuclei clearance, and LC3-associated nuclear quality control. Finally, we integrate these pathways within the tumor microenvironment and consider their implications for experimental modeling, biomarkers, and therapeutic strategies aimed at improving cancer immunotherapy.

### Literature Search Approach

Because this article is a narrative rather than a systematic review, the literature search was designed to identify mechanistically informative and clinically relevant studies rather than to perform an exhaustive quantitative evidence synthesis. Targeted searches were conducted in PubMed/MEDLINE and Google Scholar, with particular emphasis on studies published during the past decade and on the most recent literature available up to September 2026, while earlier landmark studies were retained when necessary to establish the molecular foundations of selective autophagy.

Search terms were used individually and in combination and included “selective autophagy”, “mitophagy”, “nucleophagy”, “nuclear autophagy”, “nuclear quality control”, “PINK1”, “Parkin”, “BNIP3”, “BNIP3L” or “NIX”, “FUNDC1”, “LC3”, “GABARAP”, “lamin B1”, “micronuclei”, “TEX264”, “PARP1”, “cGAS–STING”, “mitochondrial DNA”, “cancer immunity”, “tumor immune microenvironment”, “immune checkpoint blockade”, “cancer immunotherapy”, “spheroids”, and “organoids”.

Priority was given to primary mechanistic studies demonstrating cargo recognition, engagement of autophagy machinery, lysosomal delivery or degradation, and functional consequences relevant to cancer biology or tumor–immune interactions. Recent reviews were additionally used to identify emerging mechanisms and to contextualize the rapidly evolving literature. Studies were interpreted according to the strength and relevance of their evidence, with direct cancer-immunotherapy findings distinguished from established mechanistic, preclinical, emerging, or conceptual associations.

For clarity, evidence is described throughout this review using four levels. “Established mechanistic evidence” refers to studies demonstrating a defined cargo, pathway component or receptor, autophagic engagement, and functional consequence. “Preclinical cancer/immune evidence” refers to findings demonstrated in cancer cell lines, organoids, animal models, or tumor–immune experimental systems but not clinically validated. “Emerging/indirect evidence” refers to biologically relevant associations for which one or more mechanistic links remain unresolved. “Proposed/conceptual relationship” is used when the connection is supported by convergent biology but has not been directly tested in the selective autophagy context discussed.

## 2. Molecular Basis of Organelle-Selective Autophagy

Selective autophagy enables cells to recognize and eliminate defined intracellular cargos through the autophagy–lysosome system. Unlike bulk macroautophagy, which can sequester cytoplasmic material relatively non-selectively during nutrient deprivation or generalized stress, selective autophagy targets specific substrates, including damaged organelles, protein aggregates, pathogens, and nuclear or mitochondrial material [4,5,18]. This precision is particularly relevant to cancer, where malignant cells continuously encounter hypoxia, nutrient limitation, oxidative and genotoxic stress, and therapeutic injury while maintaining sufficient cellular plasticity to survive [6,7,18].

At its core, selective autophagy involves three interconnected steps: cargo recognition, recruitment of the autophagic machinery, and lysosomal degradation [4,5,18]. Cargo receptors and adaptor proteins recognize damaged or tagged cellular structures and connect them to ATG8-family proteins on forming autophagic membranes. In mammals, the ATG8 family comprises LC3 and GABARAP subfamilies, which interact with proteins containing LC3-interacting region (LIR) or related ATG8-interacting motifs [4,5,21]. This receptor–ATG8 interaction provides the molecular specificity that distinguishes selective cargo disposal from non-selective cytoplasmic recycling.

### 2.1. General Principles of Selective Autophagy

Canonical macroautophagy requires coordinated initiation, phagophore formation, ATG8 lipidation, autophagosome maturation, and lysosomal fusion [21,22]. During selective autophagy, this core machinery is spatially coupled to defined cargos through receptor- or adaptor-mediated interactions [4,5,18].

ATG8-family proteins are central to this process. Lipidated LC3 and GABARAP proteins become associated with autophagic membranes and provide docking sites for LIR-containing cargo receptors [4,5,21]. Although LC3 is commonly used as a general marker of autophagy, the mammalian ATG8 proteins are not functionally interchangeable; LC3 and GABARAP subfamilies can contribute differently to cargo recruitment, membrane dynamics, autophagosome maturation, and lysosomal fusion [5,21]. Moreover, LC3-II accumulation or increased LC3 puncta alone cannot establish increased autophagic degradation because these findings may reflect either enhanced autophagosome formation or impaired downstream turnover [23]. This distinction becomes particularly important when evaluating organelle-selective autophagy in cancer.

Cargo recognition can occur through ubiquitin-dependent or receptor-mediated, ubiquitin-independent mechanisms [4,5,18]. In ubiquitin-dependent selective autophagy, damaged substrates are tagged with ubiquitin and recognized by receptors such as p62/SQSTM1, NBR1, OPTN, NDP52, or TAX1BP1, which simultaneously interact with ATG8-family proteins. In receptor-mediated pathways, organelle-resident proteins can themselves contain LIR motifs and directly recruit autophagic membranes. BNIP3, NIX/BNIP3L, and FUNDC1 exemplify this latter mechanism during mitophagy, particularly under hypoxic or metabolic stress [24,25,26].

These routes are not merely alternative mechanisms of cellular waste disposal. They allow selective autophagy to respond differently to organelle damage, metabolic state, oxygen availability, and therapeutic stress. Consequently, the biological significance of selective autophagy depends not only on whether autophagy is activated, but also on which cargo is recognized, by which receptor, in which cellular compartment, and under which stress condition.

### 2.2. Cargo Recognition Connects Organelle Damage to Autophagic Clearance

Selective autophagy receptors function as molecular interfaces between intracellular damage and lysosomal degradation. p62/SQSTM1 is among the best-characterized soluble receptors and binds both ubiquitinated cargos and LC3 through distinct interaction domains [5,27]. Because p62 is itself degraded through autophagy, its accumulation is frequently used as an indicator of impaired autophagic turnover; however, its abundance is also transcriptionally regulated by stress and redox pathways, including KEAP1–NRF2 signaling, and therefore requires interpretation together with dynamic flux measurements [23,27].

Mitophagy illustrates particularly well how different cargo-recognition strategies can converge on the same organelle. Following mitochondrial damage, the PINK1/Parkin pathway generates ubiquitin-dependent signals that recruit receptors such as OPTN and NDP52 and engage the autophagic machinery [28,29,30]. By contrast, mitochondrial proteins including BNIP3, NIX/BNIP3L, and FUNDC1 directly interact with ATG8-family proteins through LIR motifs and can promote mitophagy independently of canonical Parkin activation [24,25,26]. Additional mechanisms involving proteins such as BCL2L13, FKBP8 and PHB2 further illustrate the mechanistic diversity of mitochondrial quality control [24,25,31]. The detailed immunological consequences of these pathways are discussed in Section 3.

Selective autophagy extends beyond mitochondria. Distinct receptor systems mediate turnover of the endoplasmic reticulum, peroxisomes, lysosomes, lipid droplets, protein aggregates, and other cellular structures [4,5,18]. These pathways reinforce a fundamental principle: selective autophagy is not a single degradative pathway but a family of cargo-specific quality-control programs.

Nuclear cargo recognition remains considerably less defined. In mammalian cells, LC3-mediated degradation of lamin B1 provides direct evidence that selected nuclear components can engage autophagy-related machinery [14,32]. Micronuclei and other DNA-containing structures add another dimension because their persistence can activate cytosolic DNA sensing, whereas their autophagic clearance may restrict the duration of such signaling. The finding that cGAS can participate in autophagic micronuclear clearance provides an important mechanistic link between nuclear quality control and innate immune regulation [18]. These nuclear processes are examined in detail in Section 4. The major mitochondrial and nuclear selective autophagy pathways discussed in this review, together with their experimental context and current level of evidence, are summarized in Table 1.

### 2.3. Selective Autophagy in Cancer Cell Adaptation

Cancer cells operate under persistent intracellular stress. Rapid proliferation increases metabolic and biosynthetic demand, oncogenic signaling produces replication and proteotoxic stress, abnormal tumor vascularization generates hypoxic and nutrient-deprived niches, and anticancer therapies add mitochondrial, genomic, and oxidative injury [6,7,18,40]. Selective autophagy can buffer these stresses by removing damaged intracellular structures before they trigger irreversible dysfunction or cell death.

Hypoxia provides a particularly relevant example. Hypoxia-responsive mitophagy pathways involving BNIP3, NIX/BNIP3L, and FUNDC1 can reduce the burden of dysfunctional mitochondria and limit mitochondrial ROS generation, thereby facilitating adaptation to oxygen deprivation [24,25,26]. From an immunological perspective, however, this adaptation may have additional consequences because mitochondrial damage is also a source of mtDNA and other danger-associated signals capable of activating innate immune pathways.

Similar principles apply during nutrient deprivation and anticancer treatment. Autophagic recycling can support metabolic homeostasis under nutrient scarcity, whereas selective disposal of therapy-damaged mitochondria and other organelles can promote survival after chemotherapy or radiotherapy [6,7,18,40]. Conversely, impaired or overwhelmed autophagic clearance may permit accumulation of organelle damage, oxidative stress, and immunogenic signals. Thus, the effect of selective autophagy cannot be classified uniformly as tumor suppressive or tumor promoting; it depends on tumor stage, cellular compartment, autophagic flux, stress intensity, and therapeutic context.

This context dependence becomes particularly important during immune attack. Cytotoxic lymphocytes expose cancer cells to inflammatory cytokines, oxidative stress, death-receptor signaling, and cytotoxic effector molecules. Efficient organelle quality control may increase the ability of tumor cells to tolerate these insults, whereas excessive clearance of damaged mitochondria or nuclear-derived material may simultaneously reduce the persistence of intracellular danger signals required for robust innate immune activation.

**Figure 1:**
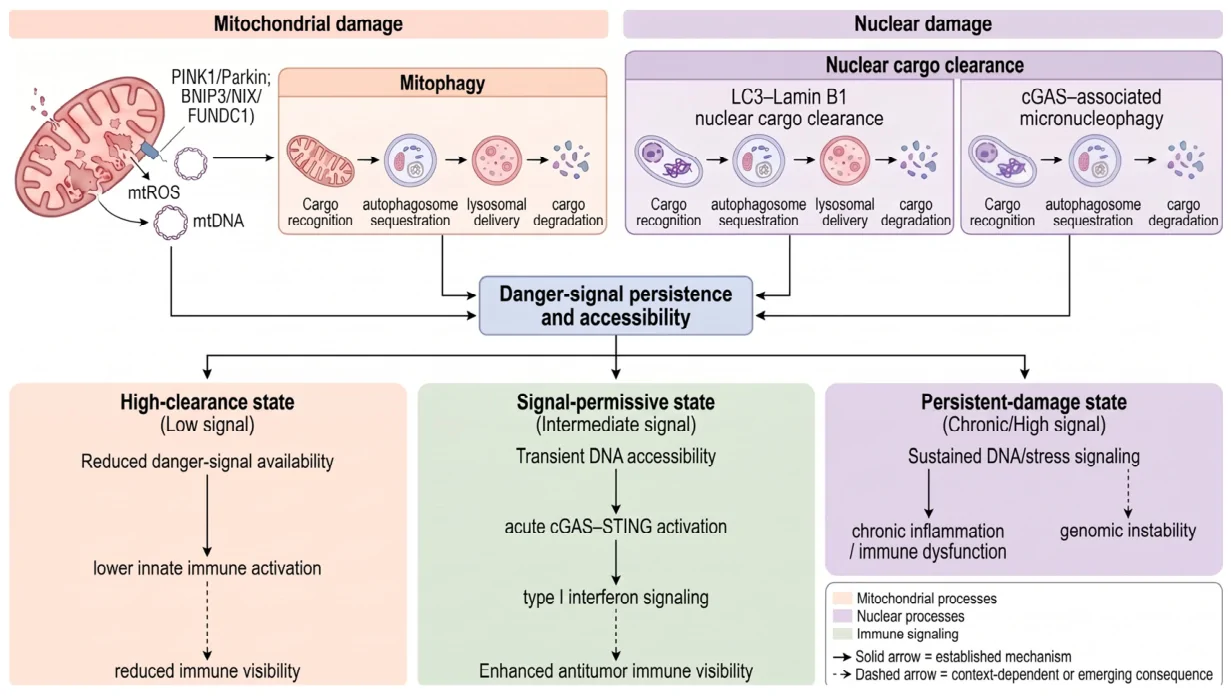
Organelle-selective autophagy as an intracellular immune-signal editing system in cancer. Mitochondrial and nuclear damage generate immunologically active intracellular signals, including mtDNA, mitochondrial ROS-associated stress, micronuclei, cytosolic chromatin, and nuclear-envelope abnormalities. Cargo-specific autophagic flux regulates the recognition, sequestration, lysosomal delivery, and degradation of these damaged structures, thereby controlling the magnitude and duration of intracellular danger-signal availability. A high-clearance state may reduce mtDNA and nuclear-derived DNA accessibility, restrain cGAS–STING signaling, and decrease tumor immunogenicity, potentially favoring immune escape. A signal-permissive intermediate state allows transient persistence of organelle-derived danger signals, supporting acute cGAS–STING activation, type I interferon signaling, antigen presentation, T-cell recruitment, and enhanced antitumor immune visibility. In contrast, persistent unresolved organelle or genomic damage may sustain inflammatory signaling, promote immune dysfunction or suppression, and contribute to genomic instability. Solid arrows indicate relationships supported by direct mechanistic evidence, whereas dashed arrows indicate emerging, indirect, context-dependent, or conceptual relationships for which direct evidence remains limited (created in BioRender. Yıldırım, B. (2026) https://BioRender.com/o2buk4x).

Selective autophagy should therefore be viewed as both a cellular stress-management system and a regulator of immune visibility. By controlling the persistence or removal of damaged mitochondria, micronuclei, and other stress-associated cargos, selective autophagy can influence whether intracellular damage remains tolerable, becomes lethal, or is translated into inflammatory signaling. This concept provides the mechanistic foundation for the mitophagy and nucleophagy pathways discussed in the following sections. This proposed relationship between organelle clearance, danger-signal persistence, and tumor immune visibility is illustrated in Figure 1.

## 3. Mitophagy in Cancer Immunity and Immunotherapy Response

Mitophagy is the selective autophagic elimination of damaged, superfluous, or dysfunctional mitochondria. In cancer, its significance extends beyond mitochondrial quality control because mitochondria also function as signaling platforms that regulate reactive oxygen species (ROS), apoptosis, metabolic adaptation, and the release of mitochondrial damage-associated molecular patterns, particularly mitochondrial DNA (mtDNA) [8,9,10,11,12,13]. Consequently, mitophagy can influence whether mitochondrial stress is neutralized intracellularly or becomes accessible to innate immune sensors. Throughout this section, we distinguish direct evidence linking mitophagy or autophagic cargo handling to immune-checkpoint regulation or antitumor immune function from mechanistic studies in which implications for immunotherapy remain inferential. Unless an immune endpoint or immunotherapy response was experimentally evaluated, such findings are described as mechanistic or emerging evidence rather than as established immunotherapy mechanisms.

This distinction is particularly relevant to cancer immunotherapy. Effective antitumor immunity depends on tumor immunogenicity, intact interferon signaling and antigen presentation, and the recruitment and persistence of cytotoxic immune cells [1,2]. Mitochondrial damage can contribute to these processes through mtDNA release, ROS accumulation, and activation of cGAS–STING and inflammasome pathways [11,12,13,41]. At the same time, persistent mitochondrial dysfunction can promote metabolic adaptation, chronic inflammatory signaling, and treatment resistance [41,42]. Mitophagy therefore lies at a critical interface between mitochondrial stress tolerance and immune visibility.

### 3.1. PINK1/Parkin-Dependent and Receptor-Mediated Mitophagy in Cancer

Two major mechanistic routes dominate mammalian mitophagy: ubiquitin-dependent PINK1/Parkin-mediated mitophagy and receptor-mediated pathways involving mitochondria-anchored LC3-interacting proteins [8,9,10]. Following mitochondrial depolarization, PINK1 accumulates on the outer mitochondrial membrane and phosphorylates ubiquitin and Parkin, promoting Parkin recruitment and amplification of mitochondrial ubiquitin signals [27,28,40]. These signals recruit autophagy receptors, particularly OPTN and NDP52, which couple damaged mitochondria to LC3/GABARAP-positive autophagic membranes [27,29]. This pathway establishes a direct molecular link between mitochondrial damage sensing and selective autophagosome formation.

The consequences of PINK1/Parkin-mediated mitophagy in cancer are context-dependent. By removing dysfunctional mitochondria, it can limit excessive ROS accumulation and genomic damage. However, established tumors may also exploit mitophagy to survive metabolic stress, chemotherapy, radiotherapy, and immune-mediated injury [8,9,42,43]. Thus, the biological outcome depends on tumor type, genetic background, mitochondrial state, autophagic flux, and treatment context.

Receptor-mediated mitophagy provides an alternative route, particularly under hypoxia and metabolic stress. BNIP3, NIX/BNIP3L, and FUNDC1 are outer mitochondrial membrane proteins containing LIR motifs that directly engage ATG8-family proteins [9,25,33,34,35]. BNIP3 and NIX are strongly associated with hypoxia-responsive mitochondrial remodeling, whereas FUNDC1 integrates hypoxic stress with mitochondrial dynamics and mitophagy [25,34,35]. These pathways may help cancer cells reduce mitochondrial mass and ROS burden in poorly oxygenated tumor regions.

Importantly, different mitophagy routes are not necessarily immunologically equivalent. PINK1/Parkin-dependent mitophagy is coupled to ubiquitin signaling and OPTN/NDP52 recruitment, whereas BNIP3/NIX/FUNDC1-mediated pathways are more directly linked to hypoxia and metabolic adaptation. The route and magnitude of mitochondrial clearance may therefore influence mtDNA release, ROS accumulation, cGAS–STING activation, and susceptibility to immune-mediated damage.

### 3.2. Mitophagy, mtDNA Release, and cGAS–STING Signaling

Mitochondrial DNA can function as a potent innate immune stimulus when released from mitochondria into the cytosol or extracellular space [11,12]. Cytosolic mtDNA can activate cGAS, leading to cyclic GMP–AMP production, STING activation, TBK1–IRF3 signaling, and induction of type I interferons and inflammatory genes [12,13,41]. In cancer, appropriately timed activation of this pathway can promote dendritic-cell activation, antigen cross-presentation, and T-cell recruitment.

Mitophagy regulates this axis by controlling the persistence of damaged mitochondria as sources of mtDNA. Efficient mitochondrial clearance can reduce mtDNA leakage and restrain cGAS–STING activation, whereas defective mitophagy may increase mitochondrial damage, mtDNA release, ROS production, and inflammatory signaling [11,12,13,41].

The immunological outcome, however, is not linear. Acute or transient cGAS–STING activation can support antitumor immunity, whereas sustained or dysregulated signaling may promote PD-L1 expression, immunosuppressive myeloid programs, T-cell dysfunction, and tumor-promoting inflammation [41,44]. Thus, mitophagy may either blunt beneficial innate immune activation or prevent maladaptive chronic inflammation depending on timing and cellular context.

This balance may become particularly important after chemotherapy or radiotherapy. Both treatments can damage mitochondria and increase mitochondrial danger signaling, but therapy-induced mitophagy can simultaneously remove damaged mitochondria and reduce oxidative stress [42,43]. In this setting, mitophagy may function as an adaptive resistance mechanism by limiting the immunogenic consequences of therapy-induced mitochondrial injury.

### 3.3. Mitophagy, ROS, and Inflammasome Regulation

Damaged mitochondria are major sources of excessive ROS. While moderate ROS levels can contribute to signaling and adaptation, persistent mitochondrial ROS can induce macromolecular damage, mitochondrial permeabilization, DNA injury, and cell death [8,11]. Mitophagy limits this burden by eliminating dysfunctional mitochondria before they become sustained sources of oxidative stress.

Mitochondrial ROS and oxidized mtDNA are also linked to activation of the NLRP3 inflammasome. Autophagy-dependent removal of damaged mitochondria can restrict mtDNA release and inflammasome activation, thereby reducing inflammatory cytokine production [45]. In tumors, however, this effect may be beneficial or detrimental depending on inflammatory context: suppression of chronic inflammasome signaling may limit tumor-promoting inflammation, whereas excessive mitochondrial clearance may also reduce inflammatory cues capable of supporting immune-cell recruitment.

The central issue is therefore not simply whether mitophagy lowers ROS, but what immunological information is lost when mitochondrial stress is removed. Cancer cells with efficient mitophagy may remain below cytotoxic oxidative thresholds and better tolerate hypoxia, treatment, and immune attack, yet the same process may reduce stress signals that would otherwise enhance tumor immunogenicity.

### 3.4. Mitophagy, Antigen Presentation, and Immune Checkpoint Regulation

A direct causal relationship between mitophagy and tumor antigen presentation remains incompletely defined. Nevertheless, mitochondrial quality control can influence several upstream processes relevant to antigen presentation, including interferon signaling, cellular metabolism, oxidative stress, and integrated stress responses [11,12,13,41]. By modulating mtDNA release and cGAS–STING activation, mitophagy may therefore indirectly affect dendritic-cell activation, antigen cross-presentation, and T-cell priming.

A more direct connection has emerged between mitochondrial quality control and PD-L1 trafficking. Xie and colleagues demonstrated that the ATAD3A–PINK1–mitophagy axis can redirect PD-L1 toward mitochondria, where mitophagic degradation reduces PD-L1 abundance and influences chemoimmunotherapy response [46]. A subsequent research highlight emphasized mitochondrial PD-L1 localization as a mechanistic link between mitophagy and immune checkpoint regulation [47]. Importantly, these findings should not be generalized to all tumor types, but they establish that mitophagy machinery can regulate immune checkpoint proteins through subcellular trafficking as well as through danger-signal control.

### 3.5. Mitophagy and Immunotherapy Resistance

Mitophagy may contribute to resistance to immune-based therapies through several complementary mechanisms. In tumor cells, efficient mitochondrial clearance can reduce mtDNA release and oxidative stress, promote survival under hypoxia and therapy-induced injury, and potentially limit innate immune activation [41,42,43]. Hypoxia-responsive pathways such as BNIP3/NIX/FUNDC1-mediated mitophagy may further cooperate with metabolic and immune-suppressive programs within the tumor microenvironment.

However, mitophagy should not be considered uniformly detrimental to cancer immunotherapy. Preserved mitochondrial quality control may limit excessive genomic and inflammatory stress, and mitophagy in immune cells can be required for metabolic fitness, persistence, and memory formation. Consequently, indiscriminate systemic inhibition of mitophagy could suppress tumor-cell adaptation while simultaneously impairing antitumor immune function.

The translational challenge is therefore to distinguish tumor-protective mitophagy from physiologically necessary mitochondrial quality control in immune and normal cells. Therapeutic strategies are more likely to succeed if they target specific mitophagy dependencies, cellular compartments, or treatment windows rather than attempting global and sustained pathway inhibition.

Overall, mitophagy represents a major regulatory interface between mitochondrial quality control, tumor adaptation, and immune signaling. Its effect on immunotherapy is determined by the balance between mitochondrial damage generation and mitochondrial clearance: insufficient clearance can produce persistent oxidative and inflammatory stress, whereas highly efficient clearance may silence danger signals that would otherwise enhance tumor recognition. This context dependency provides the foundation for interpreting mitophagy as both a potential resistance mechanism and a therapeutic vulnerability.

## 4. Nucleophagy-Related Nuclear Quality Control in Cancer

Compared with mitophagy, mammalian nucleophagy remains less comprehensively characterized but is now supported by an expanding set of mechanistically defined cargo-clearance pathways. Nucleophagy broadly refers to the selective autophagic degradation of nuclear material, including nuclear-envelope and lamina components, nuclear proteins, DNA-associated protein complexes, micronuclei, and chromatin-derived material. Recent syntheses of mammalian nucleophagy emphasize that these cargos should not be assumed to constitute a single pathway; rather, distinct receptors, adaptors, and stress conditions can engage autophagic machinery to eliminate specific nuclear substrates [20]. This distinction is particularly important in cancer, where genotoxic stress, chromosomal instability, oncogene activation, and therapy can generate structurally and biochemically diverse nuclear cargos.

This concept is particularly relevant to cancer because malignant cells frequently experience chromosomal instability, DNA damage, nuclear envelope rupture, micronucleus formation, and altered nuclear mechanics [16,17,37,48,49]. These abnormalities are not simply morphological consequences of malignant transformation. They can alter the compartmentalization of genomic DNA and expose nuclear-derived DNA to cytosolic sensors, particularly the cGAS–STING pathway [13,16,17].

Nuclear quality control may therefore influence tumor immunity by regulating the persistence and accessibility of nuclear danger signals. If damaged nuclear material persists or becomes exposed to the cytosol, innate immune signaling may increase; conversely, autophagy-related clearance of nuclear-derived material may restrict the magnitude or duration of DNA sensing. Importantly, however, such processes should not automatically be classified as canonical nucleophagy unless nuclear cargo recognition, engagement of autophagy machinery, lysosomal delivery, and degradation are experimentally demonstrated.

The distinction between cancer biology and cancer-immunotherapy evidence is particularly important for nuclear quality control. Most mammalian nucleophagy studies currently establish effects on nuclear integrity, senescence, genome stability, genotoxic stress, or treatment resistance rather than direct effects on immune-checkpoint therapy. Accordingly, throughout this section we explicitly distinguish established nucleophagy mechanisms from proposed immunological consequences.

### 4.1. Defining Mammalian Nucleophagy and Nucleophagy-Related Nuclear Quality Control

Nucleophagy is best established in yeast, where nuclear material can be degraded through piecemeal microautophagy of the nucleus and related macroautophagic processes [50,51]. These mechanisms involve coordinated interactions between nuclear membrane proteins and vacuolar or autophagy machinery, enabling selective delivery of nuclear constituents for degradation. Mammalian nuclear quality control is mechanistically more heterogeneous. Differences in nuclear envelope architecture, the nuclear lamina, chromatin organization, and lysosomal trafficking make direct extrapolation from yeast inappropriate [36]. Accordingly, the term nucleophagy should be used cautiously in mammalian cancer systems.

Several processes nevertheless support the broader concept of autophagy-dependent nuclear quality control. These include LC3-dependent degradation of nuclear lamina material, autophagic processing of nuclear-derived fragments, and clearance of damaged micronuclei [14,18,32,36,38]. These events are not necessarily components of a single pathway, but they share a common principle: nuclear-derived cargos can engage autophagy-related machinery and thereby undergo regulated degradation.

This distinction is important for tumor immunology because the immunological consequences of nuclear damage depend strongly on compartmentalization. DNA damage retained within an intact nucleus primarily engages canonical DNA-damage responses, whereas ruptured micronuclei or cytosolic chromatin can expose double-stranded DNA to cGAS and activate STING-dependent inflammatory signaling [13,16,17,37]. Nuclear quality-control mechanisms may therefore determine how long nuclear-derived danger signals remain accessible to innate immune sensors. Recent identification of TEX264-dependent clearance of trapped PARP1 has further strengthened the mechanistic definition of mammalian nucleophagy in cancer; this pathway is discussed in detail in Section 4.4 [39].

### 4.2. Nuclear Envelope Remodeling, Lamin B1 Turnover, and Autophagy

One of the strongest examples of mammalian nuclear autophagy is the selective degradation of lamin B1. Dou and colleagues demonstrated that LC3 interacts directly with lamin B1 and lamin-associated chromatin and promotes autophagic degradation of nuclear lamina material during oncogenic stress [14]. This process was associated with senescence and growth arrest, establishing a mechanistic link between autophagy, nuclear remodeling, and tumor-suppressive stress responses [14,32]. Lamin B1 is an important determinant of nuclear architecture, chromatin organization, nuclear mechanics, and cellular senescence [52,53]. Alterations in nuclear lamina organization can influence genome stability and the ability of cancer cells to tolerate mechanical deformation. During migration through confined environments, for example, nuclear deformation can cause transient nuclear envelope rupture, chromatin herniation, and DNA damage [54].

These observations raise an important distinction. Autophagic degradation of lamin B1 is experimentally established, whereas the broader proposition that adaptive nuclear-envelope remodeling functions as a general nucleophagy-based survival mechanism in cancer remains less well defined. Nuclear lamina turnover should therefore be considered a mechanistic entry point into nuclear autophagy rather than proof that all nuclear-envelope abnormalities are nucleophagic.

From an immune perspective, however, the connection is compelling. Nuclear envelope instability can increase the accessibility of genomic DNA to cytosolic sensors, either directly or through micronuclear rupture. Autophagy-related processing of damaged lamina or nuclear-derived structures may therefore influence the amount and persistence of DNA available for innate immune sensing.

### 4.3. Micronuclei, Genome Instability, and Autophagy-Linked Clearance

Micronuclei are extranuclear DNA-containing structures generated by chromosome missegregation, lagging chromosomes, chromosome fragments, replication stress, or defective mitosis [37,48,49]. They are common manifestations of chromosomal instability in cancer and can themselves become sources of additional genome damage. Micronuclear envelopes are frequently unstable. Hatch and colleagues showed that micronuclei can undergo catastrophic loss of nuclear-envelope integrity, exposing micronuclear contents and generating extensive DNA damage [37]. Subsequent work demonstrated that errors associated with micronuclei and chromosome bridges can contribute to chromothripsis and rapid genomic diversification [49].

Micronuclei also connect genome instability to innate immunity. Mackenzie and colleagues demonstrated that cGAS localizes to micronuclei generated by chromosome missegregation, while Harding and colleagues showed that mitotic progression following DNA damage enables recognition of micronuclear DNA [16,17]. Together, these studies established micronuclei as a mechanistic bridge between chromosomal instability and cytosolic DNA sensing.

Autophagy introduces an additional regulatory layer. Zhao and colleagues reported that cGAS can function as a micronucleophagy receptor and promote autophagic clearance of micronuclei in an ATG7- and ATG14-dependent manner [18]. Importantly, this clearance reduced cGAMP production, suggesting that cGAS may participate both in sensing micronuclear DNA and in limiting the persistence of the same DNA-containing structures.

More recent evidence supports the broader idea that autophagy regulates micronuclear homeostasis. In Senataxin-deficient cells, persistent R-loops were associated with cGAS-positive micronuclei, defective micronuclear membranes, and impaired autophagic clearance [38]. This study therefore provides additional evidence that defective autophagy can increase the persistence of immunologically active micronuclei. These findings suggest that autophagy can function as a nuclear danger-signal buffering mechanism. By regulating micronuclear persistence, it may restrict prolonged DNA sensing. In cancer, however, this may have opposing effects: enhanced clearance could suppress potentially beneficial antitumor innate immunity, whereas defective clearance could generate chronic inflammatory signaling and genome instability.

### 4.4. TEX264-Mediated Nucleophagy of Trapped PARP1 and Therapy Resistance

PARP inhibitors exert part of their cytotoxic activity by trapping PARP1 on chromatin, generating replication-associated lesions that are particularly toxic to homologous-recombination-deficient cancer cells. Recent evidence demonstrates that these trapped PARP1 complexes can themselves become selective nucleophagy cargos. Hoslett et al. showed that TEX264 functions as a selective autophagy receptor for trapped PARP1 and cooperates with p97/VCP and LC3 to promote its extraction from chromatin and subsequent lysosomal degradation [39].

Disruption of TEX264-mediated nucleophagy increases the persistence of trapped PARP1, promotes DNA damage and protein aggregation, and enhances cancer-cell lethality. Importantly, interference with this pathway can re-sensitize PARP-inhibitor-resistant cancer cells, establishing a direct connection between cargo-specific nucleophagy and therapeutic resistance [39].

This mechanism should nevertheless be distinguished from an established immunotherapy pathway. The available evidence directly supports a role for TEX264-dependent nucleophagy in genome protection and PARP-inhibitor resistance, but whether altered PARP1 clearance subsequently modifies cGAS–STING signaling, tumor immune visibility, or response to immune-checkpoint blockade has not yet been demonstrated. Its relevance to the framework proposed here therefore lies in establishing that mammalian cancer cells possess a receptor-defined and therapeutically consequential nuclear-cargo autophagy pathway, while its immunological consequences remain an important question for future investigation.

### 4.5. Nuclear Quality Control, cGAS–STING, and Tumor Immunogenicity

The cGAS–STING pathway provides the principal mechanistic connection between nuclear-derived DNA and tumor immunity. cGAS detects double-stranded DNA that becomes accessible outside its normal nuclear or mitochondrial compartment and catalyzes cyclic GMP–AMP production, leading to STING activation, TBK1–IRF3 signaling, and induction of type I interferons and inflammatory genes [13,44]. In appropriate contexts, this pathway promotes antitumor immunity by enhancing innate immune activation, dendritic-cell function, and T-cell responses. STING activation within tumor-associated compartments can support spontaneous and therapy-induced antitumor immunity [55]. However, chronic cytosolic DNA signaling is not uniformly beneficial. Chromosomal instability can sustain cGAS–STING and noncanonical NF-κB signaling in tumor cells and thereby promote invasion and metastasis [56]. Thus, the biological outcome depends on the duration, cellular compartment, and downstream interpretation of DNA sensing.

Autophagy-related nuclear clearance may influence this balance at multiple levels. Micronuclear degradation can limit the persistence of cGAS-accessible DNA [18,38], whereas LC3-dependent nuclear lamina degradation may alter nuclear architecture and the handling of damaged nuclear material [14,32]. These processes support a model in which nuclear autophagy functions not simply as disposal, but as a regulator of the temporal availability of DNA-derived immune signals.

Importantly, direct experimental evidence linking the kinetics of selective organelle clearance to acute-versus-chronic cGAS–STING signaling remains limited. In established experimental systems, radiation-induced cytosolic DNA can generate type I interferon-dependent antitumor signaling, whereas removal of cytosolic DNA by TREX1 attenuates this response [57]. Conversely, persistent chromosomal instability can sustain cytosolic DNA sensing and activate tumor-promoting inflammatory programs, including noncanonical NF-κB signaling [56,58]. These studies establish the biological importance of signal duration but do not by themselves demonstrate that nucleophagy determines the transition between acute and chronic STING states. For nucleophagy-related pathways, the strongest direct evidence currently shows that autophagic micronuclear clearance can reduce the persistence of cGAS-accessible substrates and cGAMP production [18]. Thus, temporal control by selective autophagy remains mechanistically plausible but incompletely resolved in cancer-immunotherapy models.

This distinction is especially important for immunotherapy. A transient burst of DNA sensing generated by therapy-induced damage may contribute to productive antitumor immunity, whereas persistent signaling from chronically unstable genomes can promote inflammatory adaptation and immune escape. The relevant question is therefore not whether nucleophagy is intrinsically immunostimulatory or immunosuppressive, but how nuclear-derived cargo clearance changes the timing, magnitude, and cellular context of cGAS–STING signaling.

### 4.6. Nucleophagy-like Remodeling Under Therapeutic Stress

Many anticancer treatments impose substantial nuclear and genotoxic stress. Chemotherapy and radiotherapy can generate DNA damage, mitotic errors, chromosome segregation defects, micronuclei, and cytosolic DNA, all of which may contribute to immunogenic stress signaling [37,48,49,59]. Autophagy-related nuclear quality control could therefore become particularly important after treatment. Removal of damaged nuclear material may limit the persistence of therapy-induced DNA sensing and support cellular recovery. Conversely, failure to eliminate unstable micronuclei or nuclear fragments may intensify innate immune signaling but also increase chromosomal instability and chronic inflammation.

This duality parallels the mitophagy paradigm described in Section 3. In both cases, selective clearance may protect cells from excessive intracellular damage while simultaneously reducing the persistence of danger signals capable of stimulating antitumor immunity. Nuclear quality control should therefore be viewed as a potential determinant of whether genotoxic stress becomes repairable, immunogenic, or evolutionarily advantageous to the tumor.

The spatial context in which these events occur is also likely to matter. Nuclear deformation, hypoxia, proliferation, DNA damage, and therapeutic exposure vary substantially across solid tumors. Three-dimensional tumor models may therefore be particularly valuable for resolving how nuclear stress and autophagic clearance vary across tumor regions.

**Figure 2:**
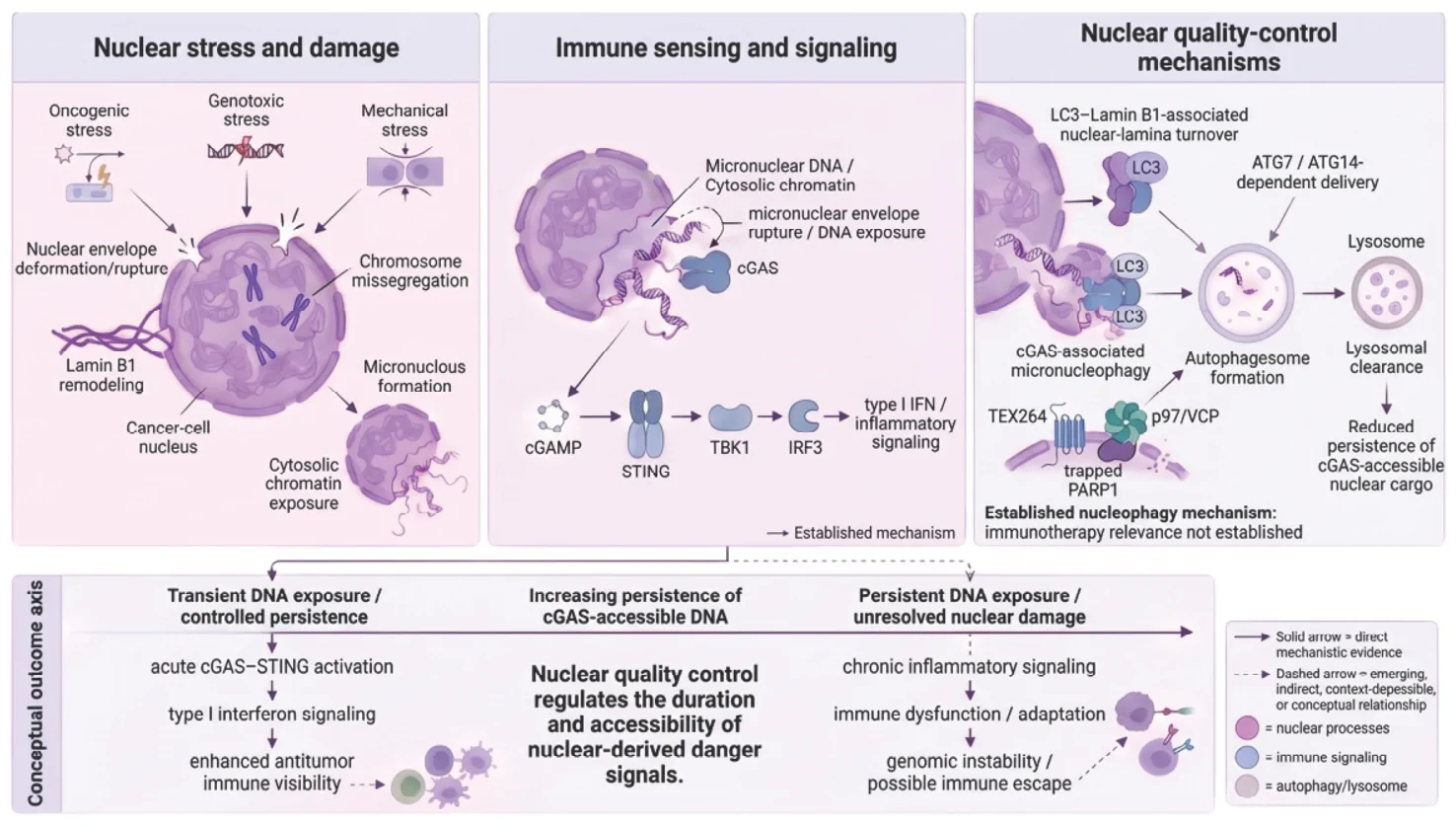
Nuclear quality control regulates the persistence of cGAS-accessible DNA in cancer. Nuclear stress arising from oncogenic, genotoxic, or mechanical insults can promote nuclear-envelope deformation or rupture, lamin B1 remodeling, chromosome missegregation, micronucleus formation, and cytosolic chromatin exposure. Rupture or instability of micronuclear envelopes can expose nuclear-derived DNA to cGAS, activating the cGAMP–STING–TBK1–IRF3 axis and downstream type I interferon and inflammatory signaling. In parallel, distinct autophagy-related nuclear quality-control mechanisms can regulate the persistence of nuclear-derived cargo. These include LC3–Lamin B1-associated nuclear-lamina turnover, cGAS-associated micronucleophagy, and TEX264–p97/VCP-mediated lysosomal clearance of trapped PARP1. The TEX264–p97/VCP–PARP1 pathway represents an established nucleophagy mechanism associated with DNA-damage responses and PARP-inhibitor resistance; however, its direct relevance to cancer immunotherapy has not yet been established. The figure emphasizes that nuclear quality control may regulate the duration and accessibility, rather than merely the presence, of nuclear-derived danger signals. Transient DNA exposure may support acute innate immune activation and enhanced antitumor immune visibility, whereas persistent DNA exposure and unresolved nuclear or genomic damage may favor chronic inflammatory signaling, immune dysfunction or adaptation, genomic instability, and possible immune escape. Solid arrows indicate relationships supported by direct mechanistic evidence, whereas dashed arrows indicate emerging, indirect, context-dependent, or conceptual relationships for which direct evidence remains limited (created in BioRender. Yıldırım, B. (2026) https://BioRender.com/dz62uba).

Overall, nucleophagy and nuclear quality control represent an emerging regulatory interface between genome instability and tumor immunity. Whereas mitophagy governs the persistence of mitochondrial danger signals, nuclear autophagy-like mechanisms can influence the fate of lamina-associated material, micronuclei, and other nuclear-derived cargos. Their importance in cancer immunotherapy will depend on whether these processes suppress immunogenic DNA sensing, prevent maladaptive chronic inflammation, or enable malignant cells to tolerate genotoxic stress. The relationship between nuclear damage, cargo clearance, cGAS–STING signaling, and the duration of nuclear-derived danger signals is summarized in Figure 2.

## 5. Interplay Between Mitophagy and Nucleophagy in Tumor Stress Adaptation

Mitophagy and nucleophagy-like nuclear quality control are often discussed separately, yet mitochondrial and nuclear stress are tightly interconnected in cancer cells. Mitochondria and the nucleus communicate through metabolic signaling, reactive oxygen species (ROS), ATP availability, stress-response pathways, and reciprocal regulation of gene expression and organelle function [60,61,62]. Consequently, hypoxia, oncogenic signaling, nutrient deprivation, chemotherapy, radiotherapy, and immune-mediated cytotoxicity frequently produce mitochondrial and nuclear damage simultaneously rather than as isolated events.

This functional coupling is particularly relevant to tumor immunity because mitochondria and the nucleus represent two major intracellular sources of immunologically active nucleic acids. Mitochondrial dysfunction can increase mtDNA release, whereas chromosomal instability, micronuclear rupture, and nuclear-envelope damage can expose genomic DNA to the cytosol [11,12,16,17,48]. Both forms of misplaced DNA can engage cGAS–STING signaling, linking organelle damage to innate immune activation. Selective autophagy therefore sits at an important regulatory intersection: by controlling the persistence of damaged mitochondria and nuclear-derived cargos, it can influence the abundance, duration, and cellular origin of danger signals available for immune sensing. Accordingly, the integration of mitochondrial and nuclear quality control presented here represents a functional framework rather than evidence that mitophagy- and nucleophagy-related pathways form a single molecular pathway.

### 5.1. Mitochondria–Nucleus Communication Under Cancer Stress

Mitochondria–nucleus communication is bidirectional. Although the majority of mitochondrial proteins are encoded by nuclear genes, mitochondrial metabolism can influence nuclear transcription, chromatin state, DNA repair, and stress responses through metabolites, ROS, energy availability, and retrograde signaling [60,61,62]. Conversely, nuclear DNA damage and stress-response pathways can alter mitochondrial biogenesis, dynamics, metabolism, apoptosis sensitivity, and autophagic turnover [61,62].

Mitophagy can therefore affect nuclear integrity indirectly. Persistent dysfunctional mitochondria increase oxidative stress, which can promote nuclear DNA damage, replication stress, telomere dysfunction, and chromosomal instability [61]. These abnormalities can in turn increase micronucleus formation and cytosolic DNA exposure [16,17,48]. Efficient mitophagy may reduce this secondary nuclear damage by limiting mitochondrial ROS, but at the same time it can reduce mtDNA-derived innate immune signals.

Nuclear stress can reciprocally reshape mitochondrial behavior. DNA damage responses influence mitochondrial metabolism and apoptotic signaling, while chromosomal instability can sustain inflammatory programs that alter metabolic state and immune interactions [56,61]. Thus, mitochondrial dysfunction can increase nuclear instability, and nuclear instability can reinforce mitochondrial stress. Selective autophagy operates within this feedback loop by determining whether damaged organelles and DNA-containing structures persist or are removed.

### 5.2. Coordinated Organelle Remodeling During Therapy-Induced Stress

Therapy-induced stress provides a particularly clear setting in which mitochondrial and nuclear quality control intersect. Chemotherapy and radiotherapy can simultaneously generate genomic damage, mitochondrial dysfunction, oxidative stress, autophagy, and the release of damage-associated molecular patterns [59,63,64].

Radiotherapy illustrates the importance of danger-signal persistence. Cytosolic DNA generated after irradiation can activate cGAS–STING and type I interferon signaling, whereas induction of the DNA exonuclease TREX1 at sufficiently high radiation doses can degrade cytosolic DNA and attenuate tumor immunogenicity [57]. Although TREX1 is not a nucleophagy mechanism, this example demonstrates a broader principle relevant to selective autophagy: the immune consequence of therapy-induced damage depends not simply on damage generation but on how long immunostimulatory material persists.

The same logic applies to mitochondrial quality control. Therapy-damaged mitochondria can generate ROS and mtDNA-derived inflammatory signals, whereas mitophagic clearance can reduce this burden and promote cellular survival. In parallel, nuclear-derived micronuclei or chromatin fragments can remain accessible to cGAS unless repaired, degraded, or otherwise removed. Coordinated mitochondrial and nuclear quality control may therefore influence whether treatment-induced stress becomes lethal, immunogenic, or compatible with tumor-cell survival.

This balance should not be interpreted as a simple relationship in which reduced autophagy necessarily improves immunogenicity. Excessive or prolonged organelle damage can produce chronic inflammatory signaling and select for stress-tolerant tumor states. Similarly, chromosomal instability can activate cGAS–STING yet ultimately support metastasis and immune adaptation rather than productive antitumor immunity [56,58]. The biological outcome therefore depends on the magnitude and timing of damage, the capacity for organelle clearance, and the downstream inflammatory program engaged.

### 5.3. Organelle-Selective Autophagy as a Determinant of Tumor Immune Visibility

The combined mitochondrial and nuclear perspective reinforces the central concept of this review: organelle-selective autophagy can act as a form of intracellular danger-signal editing. Mitophagy regulates mitochondrial danger-signal availability by controlling dysfunctional mitochondria, mtDNA release, and mitochondrial ROS. Nuclear autophagy-like mechanisms may regulate lamin-associated material, micronuclei, and other nuclear-derived cargos capable of exposing genomic DNA to innate immune sensors. Although these processes are mechanistically distinct, both determine whether intracellular damage remains compartmentalized, is degraded, or becomes immunologically accessible.

This framework expands the conventional concept of tumor immune visibility. Tumor recognition is generally discussed in terms of neoantigens, MHC expression, interferon signaling, immune checkpoints, and immune-cell infiltration. Organelle quality control adds an upstream layer by influencing whether cellular stress generates the innate signals that help initiate or sustain these immune responses.

The cellular compartment in which autophagy occurs is equally important. A pathway that promotes survival and limits danger signaling in tumor cells may simultaneously be required to preserve mitochondrial fitness and effector function in immune cells. This compartment-specific asymmetry becomes particularly important when considering therapeutic autophagy inhibition and is addressed in the following section.

Overall, the interaction between mitochondrial and nuclear quality control provides a mechanistic bridge between intracellular stress adaptation and tumor immunity. Rather than functioning as independent disposal systems, mitophagy and nucleophagy-like pathways can jointly influence the threshold at which organelle damage remains tolerable, becomes immunogenic, or evolves into chronic inflammatory adaptation.

## 6. Organelle-Selective Autophagy in the Tumor Microenvironment

The tumor microenvironment (TME) is a metabolically and immunologically heterogeneous ecosystem in which malignant, immune, stromal, and endothelial cells are exposed to hypoxia, nutrient limitation, acidosis, oxidative stress, inflammatory mediators, and treatment-induced injury. Under these conditions, autophagy is not confined to tumor-cell survival but contributes to the metabolism, stress tolerance, and function of multiple cellular compartments [65,66,67,68,69,70]. This distinction is fundamental to the interpretation of organelle-selective autophagy in cancer immunotherapy.

A pathway that allows cancer cells to withstand mitochondrial or proteotoxic stress may simultaneously be required for immune cells to maintain mitochondrial fitness, antigen-processing capacity, and sustained effector function. Consequently, the biological outcome of autophagy modulation depends not only on pathway activity but also on which cell type is using the pathway, under which microenvironmental conditions, and at what stage of the immune response.

### 6.1. Tumor-Cell Autophagy Versus Immune-Cell Autophagy

In established tumors, autophagy frequently supports adaptation to unfavorable microenvironmental conditions by maintaining metabolic substrates, limiting oxidative damage, and removing dysfunctional cellular components [66,68,70,71,72]. Autophagy can also influence tumor immune visibility more directly. A notable example is pancreatic ductal adenocarcinoma, in which Yamamoto and colleagues demonstrated that MHC-I molecules can be selectively targeted to lysosomes through an autophagy-dependent mechanism involving NBR1. Autophagy inhibition restored surface MHC-I expression, enhanced antigen presentation and T-cell responses, and reduced tumor growth in immunocompetent models [65]. This study provides direct evidence that tumor-cell autophagy can contribute to immune evasion by regulating antigen presentation.

The consequences of autophagy in immune cells can be markedly different. T lymphocytes require autophagic programs for metabolic adaptation, mitochondrial homeostasis, survival, and memory formation [73,74,75]. Dendritic cells use autophagy-related machinery in antigen processing and regulation of MHC molecules [76,77,78,79]. NK-cell persistence and memory can also depend on mitochondrial quality control, including BNIP3- and BNIP3L-dependent mitophagy [80].

**Figure 3:**
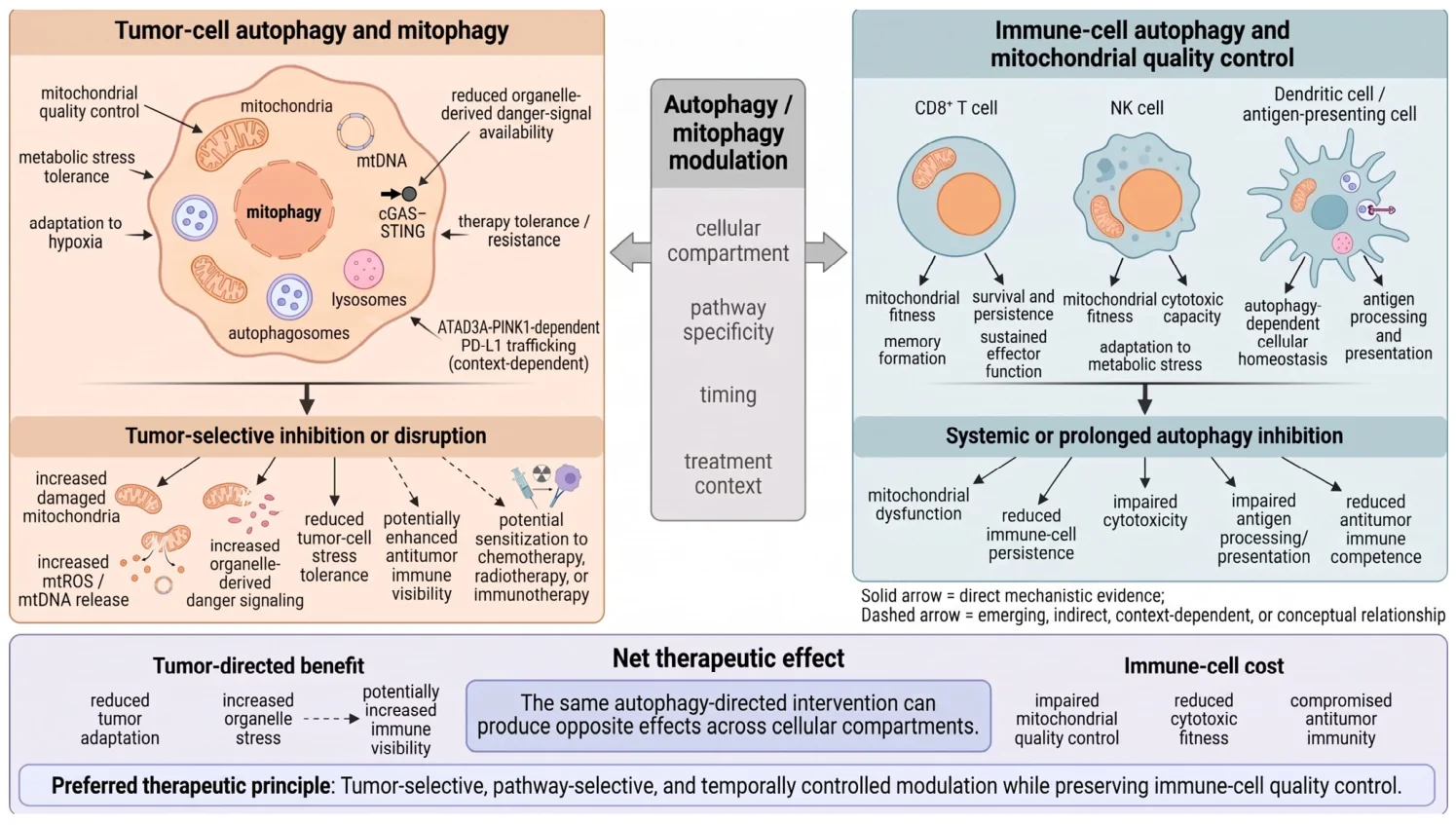
Compartment-specific consequences of autophagy modulation in the tumor microenvironment. Tumor-cell autophagy and mitophagy can support mitochondrial quality control, metabolic adaptation, reduced danger-signal availability, and therapy resistance, whereas tumor-selective disruption may increase organelle stress and potentially enhance immune visibility. In contrast, autophagy and mitochondrial quality control support the fitness and effector function of CD8^+^ T cells, NK cells, and antigen-presenting cells. Thus, systemic or prolonged autophagy inhibition may compromise antitumor immunity. The net therapeutic effect therefore depends on cellular compartment, pathway specificity, timing, and treatment context. Solid arrows indicate relationships supported by direct mechanistic evidence, whereas dashed arrows indicate emerging, indirect, context-dependent, or conceptual relationships for which direct evidence remains limited (created in BioRender. Yıldırım, B. (2026) https://BioRender.com/ti3y1bh).

This compartmental asymmetry creates a major therapeutic challenge. Global autophagy inhibition could impair a survival pathway used by cancer cells while simultaneously disrupting metabolic or functional programs required for effective antitumor immunity. The therapeutic goal should therefore not simply be to suppress autophagy, but to identify tumor-selective dependencies, cell-type-specific vulnerabilities, and treatment windows in which tumor protection can be disrupted without compromising immune competence [69,70]. These opposing consequences of autophagy modulation in tumor and immune compartments are illustrated in Figure 3.

### 6.2. T-Cell Mitochondrial Quality Control and Effector Fitness

Mitochondrial quality control is particularly important in lymphocytes exposed to the metabolic constraints of the TME. Chronic antigen stimulation, hypoxia, nutrient competition, and inhibitory signaling can disrupt mitochondrial function and contribute to T-cell exhaustion. Scharping and colleagues showed that continuous stimulation under hypoxia rapidly induces mitochondrial stress and promotes dysfunctional T-cell states, emphasizing that mitochondrial integrity is closely linked to antitumor T-cell competence [76].

Autophagy contributes to this metabolic resilience. Genetic disruption of autophagy impairs effector CD8+ T-cell survival and memory formation, while earlier studies demonstrated a requirement for autophagy in mitochondrial clearance in mature lymphocytes [73,74]. These findings support an important distinction from tumor-cell mitophagy: removal of dysfunctional mitochondria in immune cells may be necessary to preserve rather than suppress immune function.

### 6.3. NK-Cell Mitophagy and Cytotoxic-Cell Persistence

A similar principle applies to NK cells. BNIP3- and BNIP3L-mediated mitophagy is required during the contraction-to-memory transition to remove dysfunctional mitochondria and support long-lived NK-cell survival [80]. Although this evidence derives primarily from antiviral NK-cell memory rather than tumor-specific models, it demonstrates that mitophagy can be physiologically required for durable cytotoxic lymphocyte function. Metabolic adaptation is also increasingly recognized as a critical determinant of whether NK cells remain functional within nutrient-poor and immunosuppressive tumor environments [81].

### 6.4. Macrophage Mitophagy and Context-Dependent Tumor Support

Macrophages illustrate an even stronger context dependency. Mitophagy can restrain mitochondrial ROS and inflammasome activation, but in certain tumor-associated macrophage populations the same stress-adaptation machinery may favor cell survival and support tumor progression. In an ovarian cancer model, autophagic adaptation and mitophagy supported the persistence of metabolically distinct tumor-associated macrophages, whereas disruption of autophagy increased ROS-dependent macrophage loss and enhanced T-cell immunity [82]. Thus, mitochondrial quality control in immune cells should not be described simply as antitumor or protumor. Its functional outcome depends on immune-cell lineage, differentiation state, metabolic program, and the immunological role of the cell population being preserved.

### 6.5. Autophagy, Antigen Presentation, and Dendritic-Cell Function

Dendritic cells provide a direct link between intracellular quality control and adaptive antitumor immunity. Autophagy-related pathways contribute to antigen processing and presentation on MHC molecules, although their effects differ according to antigen source and presentation pathway [77,83,84]. Importantly, autophagy does not uniformly enhance antigen presentation. In dendritic cells, the ATG machinery can facilitate internalization of MHC-I molecules; loss of ATG5 or ATG7 increases surface MHC-I and can enhance CD8+ T-cell stimulation in experimental systems [78]. This finding reinforces the broader theme of this review: autophagy modifies immune signaling through multiple cargo-specific processes rather than producing a single predictable immunological outcome.

Mitochondrial fitness is also likely to influence dendritic-cell function indirectly through redox balance, inflammatory signaling, and survival under metabolic stress. However, direct evidence specifically linking organelle-selective mitophagy in tumor-associated dendritic cells to immunotherapy response remains limited. This distinction should be maintained rather than extrapolating general autophagy biology into a fully established mitophagy mechanism.

### 6.6. Nuclear Quality Control in Immune Cells: An Emerging Question

Compared with mitochondrial quality control, nucleophagy-like mechanisms in immune cells remain poorly characterized. Immune cells experience DNA damage, oxidative stress, replication stress, and cytosolic DNA sensing, but there is currently insufficient evidence to define a canonical immune-cell nucleophagy pathway analogous to well-established forms of mitophagy.

Autophagy can nevertheless influence nuclear stress indirectly by regulating mitochondrial ROS, cellular metabolism, proteostasis, and the persistence of DNA-containing structures. Likewise, cGAS–STING signaling in dendritic cells and other immune populations can promote inflammatory and type I interferon responses, while persistent DNA sensing may contribute to dysfunctional inflammatory states [13,44]. For this reason, observations such as lamin loss, micronucleus disappearance, cGAS redistribution, or LC3 puncta should not by themselves be interpreted as evidence of immune-cell nucleophagy. Demonstration of such a pathway would require identification of the nuclear cargo, engagement of autophagy machinery, lysosomal delivery, flux dependence, and a functional immune consequence.

This represents a substantial gap in the field. Whether T cells, NK cells, dendritic cells, or tumor-associated macrophages use nuclear autophagy-like quality-control pathways to modulate antitumor activity remains largely unresolved and should be treated as a future research question rather than an established mechanism.

Overall, organelle-selective autophagy in the TME is best understood as a compartment-specific regulatory system. Tumor cells may exploit autophagy to tolerate stress, alter antigen presentation, and reduce immune visibility, whereas immune cells may require related pathways to preserve metabolic fitness and effector function. This asymmetry explains why global autophagy manipulation can produce unpredictable outcomes and provides a strong rationale for tumor-selective and temporally controlled therapeutic strategies. The cell-type-specific roles of autophagy and mitochondrial quality control, together with their effects on antitumor immunity and the corresponding strength of evidence, are summarized in Table 2.

## 7. Therapeutic Implications for Cancer Immunotherapy

The therapeutic relevance of organelle-selective autophagy is supported predominantly by mechanistic and preclinical evidence. Direct clinical evidence for pharmacological manipulation of cargo-specific mitophagy or nucleophagy in combination with cancer immunotherapy remains limited. Clinical studies involving lysosomal/autophagy inhibitors or STING-directed agents therefore provide important translational context but should not be interpreted as clinical validation of a specific organelle-selective autophagy mechanism. This distinction is particularly important because broad autophagy modulation can simultaneously affect malignant, immune, stromal, and normal cellular compartments.

The central therapeutic challenge is therefore compartment and pathway specificity. In tumor cells, autophagy and mitophagy can support metabolic adaptation, eliminate damaged organelles, restrain immunogenic danger signaling, and promote resistance to therapy [3,66,67,70]. In contrast, autophagy and mitochondrial quality control may be required for the survival, metabolic fitness, and function of antitumor immune cells [73,74,75,76,77,78,79,80,81,82,83,84,85,86]. Consequently, therapeutic benefit is unlikely to result from indiscriminate activation or inhibition of autophagy.

### 7.1. Targeting Mitophagy to Enhance Antitumor Immunity

Tumor-cell mitophagy represents an attractive therapeutic target because it regulates mitochondrial ROS, mtDNA availability, metabolic adaptation, and survival after mitochondrial injury. In principle, suppressing an adaptive mitophagy pathway could increase the persistence of damaged mitochondria, augment oxidative stress and mtDNA release, and enhance innate immune signaling. However, the therapeutic outcome would depend strongly on the tumor’s baseline mitochondrial state and on whether the resulting stress generates productive antitumor immunity rather than uncontrolled inflammation or toxicity.

A major limitation is that mitophagy is mechanistically heterogeneous. PINK1/Parkin-dependent mitophagy and receptor-mediated pathways involving BNIP3, NIX/BNIP3L, or FUNDC1 are not interchangeable, and their relative importance varies according to tumor genotype, hypoxia, mitochondrial state, and treatment exposure [8,9,25,27,28,29,30]. A tumor dependent on hypoxia-induced BNIP3/NIX signaling may therefore respond differently to mitophagy modulation than a tumor relying predominantly on a PINK1/Parkin pathway.

The ATAD3A–PINK1–mitophagy axis provides a particularly relevant example of pathway-specific therapeutic modulation. In triple-negative breast cancer models, Xie and colleagues showed that paclitaxel-induced ATAD3A interferes with PINK1-dependent mitochondrial recruitment and mitophagic degradation of PD-L1, thereby favoring accumulation of PD-L1 at the plasma membrane [46]. Targeting this axis redirected PD-L1 toward mitochondria and improved chemoimmunotherapy responses in experimental systems. This work establishes that mitophagy modulation can influence immunotherapy not only through mitochondrial danger signaling but also through the intracellular trafficking and degradation of an immune-checkpoint protein [46,47].

This example also illustrates why global mitophagy inhibition may be the wrong therapeutic objective. In some settings, enhancing a particular mitophagic route could actually be beneficial if that route promotes degradation of an immune-evasion protein. Therefore, therapeutic strategies should focus on pathway-specific dependencies rather than assuming that reduced mitophagy will always enhance immunity.

### 7.2. Targeting Nuclear Quality Control: A Mechanistic Opportunity, Not Yet a Defined Drug Strategy

Therapeutic targeting of nucleophagy-like pathways is substantially less mature. Mammalian nuclear autophagy lacks a universally accepted pathway architecture or marker panel, and nuclear-derived material can be processed through overlapping systems involving DNA repair, nuclear-envelope repair, autophagy, lysosomal degradation, senescence, and innate immune sensing.

Nevertheless, nuclear quality control is therapeutically relevant because genotoxic treatments can generate micronuclei and cytosolic DNA capable of activating cGAS–STING. Radiotherapy provides a useful example: therapy-induced cytosolic DNA can promote antitumor innate immunity, whereas degradation of cytosolic DNA by TREX1 can attenuate this response [57]. This demonstrates that the persistence of nuclear-derived danger signals can materially affect treatment immunogenicity.

Autophagic clearance of micronuclei may represent another mechanism controlling this persistence. cGAS-mediated micronucleophagy can reduce cGAMP production by removing micronuclear substrates [18]. It is therefore conceivable that manipulating nuclear-cargo clearance could alter the immunogenic effects of genotoxic therapy. At present, however, there is insufficient evidence to support direct inhibition of mammalian nucleophagy as a clinically actionable strategy.

The risks are substantial. Persistent chromosomal instability and chronic cytosolic DNA signaling can promote tumor evolution, inflammatory adaptation, and metastasis [48,56]. Normal and immune cells also depend on genome-protective stress responses, although the specific contribution of receptor-defined nucleophagy in these compartments remains poorly established. Thus, nucleophagy-related mechanisms should currently be considered mechanistic and biomarker targets rather than established pharmacological targets. At present, no clinically validated strategy selectively targets mammalian nucleophagy as an anticancer or immunotherapy intervention. The TEX264–PARP1 pathway therefore represents a mechanistic and preclinical therapeutic vulnerability rather than a clinically established target [39].

### 7.3. Combining Autophagy Modulation with Immune Checkpoint Blockade

Combining autophagy modulation with immune checkpoint inhibition has a plausible biological rationale because autophagy can affect tumor-cell survival, antigen presentation, inflammatory signaling, and immune-checkpoint regulation [70,87,88]. Chloroquine and hydroxychloroquine have provided the largest clinical experience with pharmacological autophagy/lysosomal inhibition in oncology, including early-phase combination studies with immune-directed therapies. However, these compounds inhibit lysosomal function broadly and are neither organelle-selective nor autophagy-specific. Early clinical studies combining hydroxychloroquine with immune-directed regimens have demonstrated feasibility in selected settings, but efficacy has been variable and these studies do not establish that inhibition of mitophagy or nucleophagy improves immunotherapy response [89]. Consequently, clinical observations with chloroquine derivatives should be interpreted as evidence concerning broad lysosomal/autophagy modulation rather than cargo-specific selective autophagy.

Preclinical observations nevertheless provide compelling mechanistic rationales for more selective combinations. In pancreatic cancer, tumor-cell autophagy promotes MHC-I degradation and reduced antigen presentation [65]. In another setting, the ATAD3A–PINK1 pathway controls mitochondrial trafficking and degradation of PD-L1 [46]. These examples demonstrate that specific autophagic cargos and regulatory nodes can directly influence immune recognition.

Recent reviews similarly emphasize reciprocal regulation between autophagy and immune checkpoints, but also highlight the difficulty of predicting whether pathway activation or inhibition will be beneficial across tumor contexts [88,90]. The autophagy–checkpoint relationship should therefore be considered bidirectional rather than reduced to a simple model in which autophagy inhibition sensitizes every tumor to PD-1/PD-L1 blockade.

A rational combination strategy should satisfy three criteria. First, the targeted tumor should show evidence of an autophagy-dependent immune-evasion mechanism or therapy-induced adaptive pathway. Second, the intervention should preserve the autophagy or mitochondrial quality-control programs required by antitumor immune cells whenever possible. Third, pathway modulation should be demonstrated using dynamic flux measurements rather than inferred from static LC3 or p62 abundance [24].

### 7.4. STING Modulation and Organelle-Derived Danger Signaling

The convergence of mitochondrial and nuclear stress on cGAS–STING creates another therapeutic opportunity. STING agonists have been developed to enhance innate immune activation and convert poorly inflamed tumors into environments more permissive to adaptive immune responses. Several classes of STING agonists have entered clinical development, frequently in combination with other immunotherapies [91]. Clinical translation has nevertheless been challenging. Early-phase studies of intratumoral STING agonists, including MIW815/ADU-S100 and ulevostinag/MK-1454, have demonstrated pharmacodynamic activity and manageable toxicity with limited or context-dependent antitumor activity across early-phase studies [92,93]. These trials establish the clinical tractability of STING-directed modulation but do not demonstrate that manipulation of mitophagy- or nucleophagy-related cargo clearance improves STING-targeted therapy.

However, the relationship between organelle-selective autophagy and STING therapy is unlikely to be straightforward. A tumor that efficiently eliminates damaged mitochondria or micronuclei may generate relatively little endogenous cytosolic DNA, whereas a genomically unstable tumor may already display sustained STING signaling. The therapeutic value of additional STING activation may therefore depend on baseline pathway integrity, cytosolic DNA burden, and the immunological consequences of pre-existing signaling.

Recent analyses emphasize this context dependency: STING activation can promote dendritic-cell maturation, type I interferon production, and immune-cell recruitment, yet excessive or prolonged activation may contribute to immune exhaustion, myeloid reprogramming, or treatment-limiting inflammation [91,94]. Organelle-selective autophagy may therefore become relevant as a biomarker of endogenous STING substrate availability. Measurements of mtDNA leakage, micronucleus burden, mitochondrial damage, cGAS localization, and autophagic flux could potentially identify tumors in which innate immune activation is being generated, buffered, or chronically sustained.

### 7.5. Biomarker-Guided and Compartment-Resolved Therapeutic Strategies

The near-term clinical value of organelle-selective autophagy may therefore lie as much in patient stratification and pharmacodynamic monitoring as in direct pathway inhibition. For mitophagy, potentially informative measurements include PINK1 stabilization, phospho-ubiquitin, Parkin or OPTN/NDP52 recruitment, BNIP3/NIX/FUNDC1 expression, mitochondrial damage, mtDNA release, mitochondrial ROS, and direct mitophagy reporters. For nuclear quality control, candidate readouts include lamin B1 remodeling, nuclear-envelope abnormalities, micronucleus burden, cGAS-positive micronuclei, cytosolic chromatin, and downstream STING activation.

Because no single marker establishes selective autophagic activity or its immune consequence, biomarker strategies should integrate cargo damage, pathway engagement, dynamic flux, innate immune signaling, and cellular compartment. Spatial and single-cell approaches may be particularly useful for distinguishing tumor-cell programs from autophagy occurring in lymphoid, myeloid, stromal, or other tumor-associated populations.

**Figure 4:**
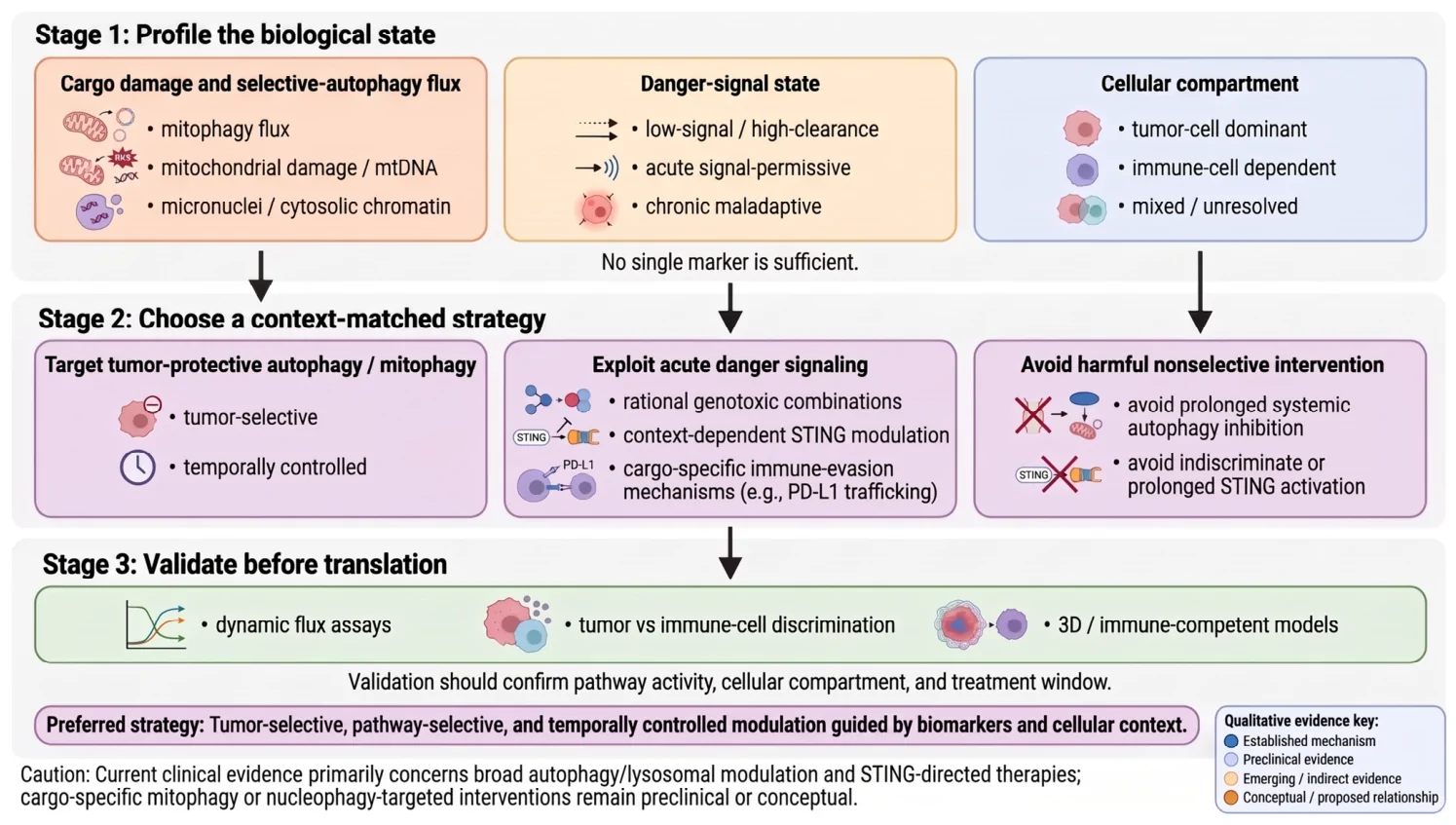
Biomarker-guided therapeutic modulation of organelle-selective autophagy in cancer immunotherapy. The proposed framework integrates cargo damage and selective autophagy flux, danger-signal state, and cellular compartment to guide context-matched therapeutic strategies. Potential approaches include tumor-selective and temporally controlled targeting of tumor-protective autophagy or mitophagy, exploitation of acute danger signaling, and avoidance of prolonged nonselective autophagy inhibition or indiscriminate STING activation. Translation requires dynamic flux measurements, discrimination between tumor- and immune-cell compartments, and validation in 3D or immune-competent models. Evidence levels range from established mechanisms to preclinical, emerging/indirect, and conceptual/proposed relationships; current clinical evidence primarily concerns broad autophagy/lysosomal modulation and STING-directed therapies rather than cargo-specific mitophagy- or nucleophagy-targeted interventions (created in BioRender. created in BioRender. Yıldırım, B. (2026) https://BioRender.com/w3lzg4s).

Taken together, the therapeutic objective should not be indiscriminate autophagy suppression. A more plausible strategy is tumor-selective, pathway-selective, and temporally controlled modulation of organelle quality control, guided by evidence that a defined autophagic program is protecting the tumor, suppressing productive danger signaling, or regulating an actionable immune-evasion mechanism. The main therapeutic strategies, their tumor rationale, potential immune-cell consequences, and current level of evidence are summarized in Table 3. A biomarker-guided framework for selecting and validating context-specific interventions is presented in Figure 4.

## 8. Experimental Models and Methodological Considerations

Understanding organelle-selective autophagy in cancer immunotherapy requires experimental systems capable of resolving both intracellular cargo turnover and its consequences for tumor–immune interactions. This is challenging because selective autophagy is dynamic, compartment-specific, and highly sensitive to metabolic and therapeutic context. Static changes in LC3, p62/SQSTM1, PINK1, Parkin, lamin B1, mitochondrial mass, or micronucleus abundance cannot by themselves demonstrate productive autophagic degradation [24,95]. Robust experimental designs should therefore combine evidence of cargo recognition, lysosomal delivery, dynamic flux, and functional consequences.

Model selection is equally important. Solid tumors contain spatial gradients of oxygen, nutrients, pH, mechanical stress, immune infiltration, and drug exposure that influence mitochondrial function, nuclear integrity, autophagic flux, and innate immune signaling. Reductionist 2D systems remain indispensable for mechanistic dissection, but conclusions concerning tumor adaptation or immunotherapy should be validated in models that better reproduce this spatial heterogeneity.

### 8.1. From Two-Dimensional Cultures to Three-Dimensional Tumor Models

Two-dimensional cultures remain valuable for causal pathway dissection but poorly reproduce the spatial gradients of oxygen, nutrients, mechanics, and drug exposure characteristic of solid tumors. These variables are particularly relevant to organelle-selective autophagy because hypoxia and metabolic stress regulate mitophagy, whereas mechanical and genotoxic stress influence nuclear-envelope integrity, micronucleus formation, and nuclear-cargo processing [16,17,25,37,54,96,97].

Spheroids, patient-derived organoids, and tumor–immune co-culture systems can therefore complement 2D models by introducing spatial and multicellular complexity [98,99,100,101,102,103,104,105,106]. The optimal strategy is model layering: mechanisms should first be resolved in tractable systems and then validated in models that reproduce the relevant tumor microenvironmental constraint. The complementary strengths, limitations, and recommended applications of the main experimental models discussed here are summarized in Table 4.

### 8.2. Demonstrating Mitophagic Flux

Mitophagy should not be inferred from mitochondrial loss or mitochondria–LC3 colocalization alone. Convincing evidence requires pathway engagement together with lysosomal delivery and cargo degradation. PINK1 stabilization, phospho-ubiquitin, Parkin or OPTN/NDP52 recruitment, and BNIP3/NIX/FUNDC1 expression can establish pathway engagement, whereas pH-sensitive reporters such as mt-Keima and mito-QC provide stronger evidence of mitochondrial delivery to acidic lysosomal compartments [10,24,95,107,108,109]. Genetic perturbation, lysosomal inhibition, rescue experiments, and time-course analyses remain important for establishing specificity.

### 8.3. Establishing Nucleophagy-like Nuclear Cargo Degradation

Mammalian nucleophagy-related processes require particularly stringent validation. Reduced lamin B1, micronucleus disappearance, LC3 puncta, or cGAS localization alone cannot establish selective nuclear-cargo degradation because these changes may result from senescence, mitosis, cell death, nuclear-envelope repair, or alternative processing mechanisms [37,52,97]. Evidence should therefore identify the nuclear cargo, demonstrate engagement of autophagy machinery and lysosomal degradation, and establish pathway dependence. LC3-dependent lamin B1 degradation, cGAS-associated micronuclear clearance, and TEX264-mediated clearance of trapped PARP1 provide increasingly well-defined examples [14,18,39]. Ultrastructural and high-resolution imaging can support cargo localization and membrane relationships but should be combined with dynamic, genetic, and lysosomal evidence because morphology or colocalization alone does not establish flux [24,95,110,111,112].

### 8.4. Linking Organelle Turnover to Immune Function

Demonstration of selective cargo degradation should be coupled to functional immune endpoints relevant to the proposed mechanism. Depending on the pathway, these may include mtDNA release, cGAS–STING activity, type I interferon signaling, MHC-I abundance, PD-L1 trafficking, dendritic-cell activation, T- or NK-cell cytotoxicity, cytokine production, and immune-cell infiltration. Tumor-cell pathway activity should, whenever possible, be distinguished from autophagy occurring in immune and stromal compartments.

Temporal resolution is essential because organelle damage, danger-signal release, innate sensing, and autophagic clearance can occur sequentially. Time-course and live-cell approaches are therefore preferable to single endpoints when testing whether selective autophagy changes the duration of immune signaling. Key experimental readouts, their principal limitations, and the validation required to establish organelle-selective autophagic flux are summarized in Table 5.

## 9. Current Gaps and Future Directions

Despite growing interest in organelle-selective autophagy, major conceptual, methodological, and translational gaps remain. Mitophagy has a comparatively mature mechanistic framework, whereas mammalian nucleophagy comprises a growing but still heterogeneous collection of cargo-specific nuclear quality-control mechanisms. Recent receptor-defined mechanisms, including TEX264-dependent clearance of trapped PARP1, strengthen the mechanistic basis of mammalian nucleophagy but do not imply that all forms of lamin turnover, micronuclear clearance, or chromatin processing constitute a unified pathway [20,39]. Autophagy-dependent lamin B1 degradation, micronuclear clearance, and cGAS-associated micronucleophagy provide compelling proof-of-concept evidence, but these observations do not yet constitute a unified cancer-associated nucleophagy pathway [14,18,36].

The next phase of the field should therefore move beyond asking whether nuclear-derived material can undergo autophagic processing and instead determine which nuclear cargos are recognized, by which receptors or adaptors, under which stresses, and with what immunological consequences.

### 9.1. Establishing a Mechanistic Definition of Mammalian Nucleophagy

A major unresolved issue is terminology. In mammalian cancer studies, the term nucleophagy is sometimes applied broadly to processes that may be mechanistically distinct, including LC3-dependent lamin B1 degradation, micronuclear clearance, and processing of cytosolic chromatin.

Future studies should define nucleophagy using criteria analogous to those applied to other selective autophagy pathways: identification of the nuclear-derived cargo, demonstration of selective recruitment of autophagy machinery, evidence of lysosomal delivery and degradation, and genetic or pharmacological demonstration of pathway dependence. Whenever possible, the relevant receptor or adaptor should also be identified.

This distinction is more than semantic. Without mechanistic criteria, changes in lamin B1, micronucleus abundance, or LC3 localization risk being interpreted as nucleophagy even when they reflect senescence, nuclear-envelope repair, cell division, cell death, or other stress responses. The field therefore requires functional definitions rather than morphology-based labels.

### 9.2. Resolving When Organelle Clearance Promotes or Suppresses Tumor Immunity

A key unresolved issue is how the magnitude and duration of organelle-derived danger signaling determine immune outcome. Acute DNA sensing can support type I interferon-dependent antitumor responses, whereas persistent signaling in chromosomally unstable tumors can support maladaptive inflammatory programs [56,57,58]. Future studies should therefore resolve how cargo-specific organelle clearance changes the kinetics, not merely the presence, of innate immune signaling.

### 9.3. Distinguishing Tumor-Cell and Immune-Cell Autophagy

One of the most important unresolved translational issues is compartment specificity. Tumor-cell autophagy can promote stress tolerance, influence antigen presentation, and reduce the persistence of organelle-derived danger signals, whereas autophagy and mitophagy in immune cells may be required for metabolic fitness, memory formation, and sustained effector function [65,70,73,76,80].

Future studies should prioritize cell-type-specific perturbation and spatially resolved analysis because systemic pathway manipulation can affect malignant and immune compartments in opposing ways. Therapeutic strategies should therefore be evaluated according to the cell population, cargo dependency, and treatment window rather than bulk pathway activity alone.

### 9.4. Moving from Static Markers to Integrated Functional Biomarkers

Biomarker development should instead integrate several layers of information: organelle damage, cargo recognition, lysosomal turnover, innate immune signaling, antigen presentation, and tumor–immune composition. Spatial and single-cell technologies may be particularly valuable for determining whether a selective autophagy signature originates from malignant cells, immune cells, or specific hypoxic and therapy-resistant tumor niches.

Such integrated signatures could ultimately help stratify tumors for immune checkpoint blockade, radiotherapy–immunotherapy combinations, DNA damage response inhibitors, or STING-targeted strategies. However, prospective validation will be necessary before any organelle-selective autophagy marker can be considered clinically predictive.

### 9.5. Improving Experimental Models of Organelle–Immune Interactions

Future studies should combine mechanistic perturbation with physiologically relevant 3D and immune-competent systems capable of resolving spatially heterogeneous organelle stress and tumor–immune interactions [98,99,100,101,102,103,104,105,106]. Such models should complement rather than replace reductionist systems and in vivo validation.

### 9.6. Translating Organelle-Selective Autophagy into Therapeutic Strategies

Therapeutically, the central challenge is specificity. Broad autophagy inhibition affects lysosomal and metabolic processes in both malignant and non-malignant cells and is unlikely to reproduce the effects of selectively disrupting a defined mitophagy or nuclear-cargo pathway.

More promising approaches may include targeting tumor-specific mitophagy dependencies, manipulating cargo-specific immune-evasion mechanisms, temporally coordinating autophagy modulation with genotoxic therapies, or using organelle-stress signatures to guide STING- or checkpoint-based treatment. Recent reviews increasingly emphasize this need for pathway- and compartment-specific strategies rather than uniform autophagy suppression [90,113,114].

The same caution applies to nuclear quality control. Direct pharmacological targeting of mammalian nucleophagy remains premature because the relevant mechanisms, receptors, and normal-tissue functions are incompletely resolved. In the near term, nucleophagy-related processes are likely to be more valuable as mechanistic biomarkers and experimental targets than as standalone drug targets.

Overall, the field should now move from descriptive observations toward mechanistic and functional integration. The critical question is no longer simply whether mitophagy or nucleophagy occurs in cancer, but how selective organelle clearance determines the immunological fate of cellular stress. Resolving this question will require cargo-specific mechanistic definitions, compartment-resolved models, dynamic measurements, and therapeutic strategies capable of separating tumor-protective quality control from the autophagic programs required for effective antitumor immunity.

## 10. Conclusions

Organelle-selective autophagy represents an important regulatory layer linking intracellular stress management to tumor immunity. Cancer cells continuously encounter metabolic, oxidative, genotoxic, therapeutic, and immune-mediated stress, and the immunological consequences of these insults depend in part on how damaged organelles and organelle-derived danger signals are processed.

Mitophagy is currently the best-characterized example of this principle. By regulating mitochondrial turnover, ROS accumulation, mtDNA release, and metabolic adaptation, mitophagy can influence both tumor-cell survival and innate immune signaling. Its effects are inherently context-dependent: efficient mitochondrial clearance may protect cells from excessive damage and chronic inflammation, but it can also reduce the persistence of mitochondrial danger signals that would otherwise enhance tumor immunogenicity.

Nucleophagy and related nuclear quality-control mechanisms are less well defined but extend the same conceptual framework to nuclear-derived stress. Autophagy-dependent lamin B1 degradation, micronuclear clearance, and cGAS-associated processing of nuclear-derived DNA suggest that nuclear material can be actively regulated rather than functioning solely as a passive consequence of genomic instability. These pathways may therefore influence the magnitude and duration of cytosolic DNA sensing and cGAS–STING signaling.

Taken together, mitophagy and nucleophagy-like pathways support a broader view of organelle-selective autophagy as a form of intracellular immune-signal editing. By controlling the persistence, accessibility, and degradation of mitochondrial and nuclear danger signals, selective autophagy can influence whether cellular stress is translated into productive antitumor immunity, adaptive stress tolerance, or chronic inflammatory states that favor tumor progression.

The translational challenge is specificity. Autophagy can protect malignant cells while simultaneously supporting mitochondrial fitness, antigen presentation, and effector function in immune cells. Therapeutic strategies should therefore prioritize tumor-selective, pathway-selective, and temporally controlled modulation rather than indiscriminate autophagy inhibition. Similarly, nucleophagy-related mechanisms currently appear more suitable as mechanistic and biomarker targets than as established pharmacological targets.

Future progress will depend on rigorous demonstration of organelle-specific cargo recognition and degradation, dynamic measurements of autophagic flux, compartment-resolved analysis of tumor and immune cells, and experimental systems that preserve the spatial complexity of the tumor microenvironment. Integrating these approaches may clarify when organelle damage should be preserved, amplified, or eliminated to improve immunotherapy response.

Ultimately, the value of organelle-selective autophagy in cancer immunotherapy lies not simply in whether damaged organelles are removed, but in how that removal changes the immunological meaning of cellular stress. Defining this balance may reveal new biomarkers of resistance and provide a rationale for therapeutic strategies that convert organelle stress into effective antitumor immunity.

## Figures and Tables

**Table 1:** Organelle-selective autophagy pathways relevant to tumor–immune regulation and strength of supporting evidence.

Pathway	Cargo/Key Machinery	Tumor Type or Model	Cellular Compartment	Immune/Therapeutic Endpoint	Evidence Level	Representative References
PINK1/Parkin-dependent mitophagy	Damaged mitochondria; PINK1, Parkin, p-Ub, OPTN/NDP52, LC3/GABARAP	Multiple mammalian cancer models; mitochondrial or therapy-induced stress	Primarily tumor-cell mitochondria	Controls mitochondrial quality and mtDNA/mtROS availability; potential modulation of cGAS–STING and tumor immune visibility	Established mechanistic evidence; cancer-immune consequences context-dependent	[8,9,10,28,29,30]
BNIP3/NIX/FUNDC1-mediated mitophagy	Mitochondria; BNIP3, NIX/BNIP3L, FUNDC1–LC3/GABARAP	Hypoxic and metabolically stressed solid-tumor models	Primarily tumor-cell mitochondria; selected immune-cell contexts for BNIP3/BNIP3L	Supports mitochondrial adaptation and can alter organelle-derived danger-signal availability	Established mechanistic evidence; immunotherapy relevance emerging	[24,25,26,33,34,35]
Alternative receptor-mediated mitophagy	Mitochondria or mitochondrial substructures; BCL2L13, FKBP8, PHB2	Mechanistic mammalian/cancer-cell models	Mitochondria	Provides additional cargo-specific routes of mitochondrial clearance; direct immune endpoint not established	Established/strong mechanistic evidence; cancer-immune role limited	[24,25,31]
LC3-dependent nuclear-lamina turnover	Lamin B1 and lamin-associated chromatin; LC3 and core autophagy machinery	Activated-RAS/oncogenic-stress and senescence models in mammalian cells	Nuclear lamina/nuclear-derived cargo	Regulates nuclear-lamina turnover and stress adaptation; direct cancer-immunotherapy consequence not established	Established mechanistic evidence; immune relevance emerging	[14,32]
cGAS-associated micronucleophagy	Micronuclei; cGAS, ATG7, ATG14 and autophagic machinery	Genotoxic-stress and micronucleus-forming mammalian cell models	Micronuclei/cytosolic nuclear-derived cargo	Promotes micronuclear clearance and reduces persistence of cGAS-accessible DNA/cGAMP production	Direct mechanistic evidence; cancer-immunotherapy relevance emerging	[18]
Autophagic processing of nuclear-derived chromatin	Cytosolic chromatin/nuclear fragments; incompletely defined LC3/ATG-dependent machinery	Nuclear-envelope instability, mitotic and genotoxic-stress models	Nuclear-derived/cytosolic chromatin	May alter persistence of cytosolic DNA and downstream DNA sensing	Emerging/indirect evidence; should not automatically be termed canonical nucleophagy	[36,37,38]
TEX264/p97-mediated nucleophagy	Chromatin-trapped PARP1; TEX264, p97/VCP, LC3 and lysosomal machinery	HR-deficient, particularly BRCA1-deficient, and PARPi-resistant cancer models	Chromatin-associated nuclear cargo	Clears trapped PARP1, limits PARPi-induced DNA damage and promotes PARPi resistance; direct immune endpoint not established	Established receptor-defined nucleophagy mechanism; direct preclinical therapeutic-resistance evidence; immunotherapy relevance not established	[39]

**Table 2:** Cell-type-specific roles of autophagy and mitochondrial quality control in the tumor–immune microenvironment.

Cell Type	Role of Autophagy/Mitophagy	Effect on Antitumor Immunity	Strength of Evidence	Representative Refs
Tumor cells	Stress tolerance, organelle quality control; autophagy-dependent MHC-I degradation in selected tumors	May reduce immune visibility and promote therapy resistance	Direct preclinical tumor–immune evidence	[65,70]
CD8+ T cells	Mitochondrial homeostasis, survival, metabolic fitness and memory	Generally supports sustained antitumor effector function	Established immune-cell biology; tumor-context evidence supportive	[73,74,75,76]
NK cells	BNIP3/BNIP3L-dependent mitochondrial clearance and memory-cell fitness	Supports cytotoxic-cell persistence; direct cancer-specific evidence more limited	Established mechanistic immune-cell evidence; cancer relevance emerging	[80,81]
Dendritic cells	Autophagy-related antigen processing and regulation of MHC trafficking	Context-dependent effects on antigen presentation and T-cell priming	Mechanistic/preclinical evidence	[77,78,79,83,84]
Tumor-associated macrophages	Mitochondrial stress control and metabolic adaptation	Can preserve macrophage populations that support tumor progression in selected models	Preclinical, context-dependent	[82,85,86]
Immune-cell nuclear quality control	Nuclear stress handling; receptor-defined nucleophagy not established in tumor-infiltrating immune cells	Immunological consequence unresolved	Emerging/conceptual	—

**Table 3:** Therapeutic strategies targeting organelle-selective autophagy in cancer immunotherapy.

Therapeutic Strategy	Tumor Rationale	Immune-Cell Concern	Rational Combination	Evidence Level
Global autophagy/lysosomal inhibition	Increases metabolic and organelle stress; may impair tumor adaptation	May compromise T-cell, NK-cell and APC function	Chemotherapy, radiotherapy, ICB	Clinical experience; organelle-selective relevance indirect
Tumor-selective mitophagy inhibition	Increases mitochondrial stress and may preserve mtDNA/mtROS danger signaling	Systemic inhibition may impair immune-cell mitochondrial fitness	Chemotherapy, radiotherapy, ICB	Preclinical/mechanistic
Cargo-specific immune-evasion targeting	Targets defined autophagy-linked immune-evasion mechanisms, e.g., ATAD3A–PINK1–PD-L1 trafficking	Immune-cell consequences depend on pathway specificity	ICB ± chemotherapy	Strong mechanistic/preclinical in selected contexts
Acute danger-signal/cGAS–STING exploitation	Enhances transient DNA sensing and tumor immune visibility	Excessive or chronic signaling may become maladaptive	Radiotherapy, DDR inhibitors, ICB	Clinical development for STING; autophagy linkage mainly mechanistic
Nuclear-cargo clearance modulation	May prolong cGAS-accessible micronuclear/nuclear-derived DNA	Risk of genomic instability, inflammation and normal-cell toxicity	Genotoxic therapy/DDR inhibition	Exploratory/preclinical
Biomarker-guided selective autophagy targeting	Enriches for tumors with actionable autophagy dependencies	Helps avoid interventions that compromise immune competence	Pathway-specific combinations	Translational priority; no validated biomarker panel
Spatially/temporally controlled modulation	Restricts inhibition to tumor or treatment windows where autophagy is protective	May preserve immune-cell quality control outside the intervention window	Radiotherapy, chemotherapy, ICB, STING-directed therapy	Conceptually promising; limited clinical validation

**Table 4:** Experimental models for resolving organelle-selective autophagy in tumor–immune contexts.

Model	Main Value	Main Limitation	Recommended Use
2D monolayer	Controlled mechanistic dissection	Poor spatial realism	Establish causality; validate key findings in 3D/in vivo
Tumor spheroids	Hypoxia, nutrient and drug gradients	Size/diffusion variability	Spatially resolved autophagy and damage analysis [98,99,100,101]
Patient-derived organoids	Patient-specific architecture and genotype	Variable immune/stromal content	Pair with matched tissue or immune co-culture [102,103]
Tumor–immune co-culture	Direct tumor–immune interaction	Incomplete TME complexity	Resolve autophagy separately in tumor and immune cells
Functional immune assays	T/NK killing, cytokines, antigen presentation	Does not establish selective autophagy mechanism	Couple with cargo-specific flux and genetic perturbation

**Table 5:** Experimental readouts for establishing organelle-selective autophagic flux.

Readout	What It Indicates	Key Limitation	Essential Validation
LC3-II/LC3 puncta	Autophagosome abundance	Does not establish flux	Lysosomal blockade + dynamic flux assay
p62/SQSTM1	Autophagy-associated cargo turnover	Transcriptionally regulated; nonspecific alone	Turnover analysis + complementary flux markers
Mitochondria–LC3 colocalization	Mitochondrial sequestration	Does not prove lysosomal degradation	Lysosomal colocalization + genetic controls
PINK1/p-Ub/Parkin	PINK1–Parkin pathway engagement	Not applicable to all mitophagy routes	Mitochondrial degradation or direct flux reporter
BNIP3/NIX/FUNDC1	Receptor-mediated mitophagy engagement	Expression ≠ mitophagic flux	Receptor perturbation + lysosomal mitochondrial turnover
mt-Keima/mito-QC	Mitochondrial delivery to lysosomes	Reporter/model-dependent	Pathway-specific genetic validation
Lamin B1 loss/LC3–Lamin B1 association	Nuclear-lamina remodeling/cargo recognition	Does not alone prove autophagic degradation	ATG dependence + lysosomal degradation + time course
Micronucleus burden/cGAS+ micronuclei	Nuclear damage and DNA accessibility to cGAS	Does not prove clearance or productive STING signaling	Envelope integrity + cGAMP/STING readouts + ATG-dependent clearance
Transmission electron microscopy (TEM)	Ultrastructure of autophagic membranes, mitochondria, nuclear-envelope abnormalities, and other organelle remodeling	Static morphology; cannot establish selective autophagic flux or pathway identity alone	Combine with cargo-specific molecular markers, lysosomal/flux assays, genetic perturbation, and time-course analysis [24,95,110,111,112]

## Data Availability

No new data were created or analyzed in this study. Data sharing is not applicable to this article.

## Abbreviations

The following abbreviations are used in this manuscript:

ATG	autophagy-related gene/protein
BNIP3	BCL2 interacting protein 3
cGAS	cyclic GMP–AMP synthase
FUNDC1	FUN14 domain-containing protein 1
GABARAP	GABA type A receptor-associated protein
ICB	immune checkpoint blockade
LC3	microtubule-associated protein 1 light chain 3
MHC-I	major histocompatibility complex class I
mtDNA	mitochondrial DNA
NIX	BCL2 interacting protein 3-like
NK	natural killer
PD-1	programmed cell death protein 1
PD-L1	programmed death-ligand 1
PINK1	PTEN-induced kinase 1
ROS	reactive oxygen species
STING	stimulator of interferon genes
TEM	transmission electron microscopy
TME	tumor microenvironment
PARP1	poly(ADP-ribose) polymerase 1
PARPi	poly(ADP-ribose) polymerase inhibitor
TEX264	testis-expressed protein 264
VCP	valosin-containing protein
